# Supplementary material for: A systematic review of population-based studies on lipid profiles in Latin America and the Caribbean
Source: eLife. 2020 Aug 18;9:e57980. doi: 10.7554/eLife.57980 (PMC7434333; doi:10.7554/eLife.57980)
Supplement: Supplementary file 1. [file elife-57980-supp1.docx]

**A systematic review of population-based studies on Lipid profiles in Latin America and the Caribbean**

**Contents**

[PRISMA 2009 Checklist 3](#_Toc42494716)

[Search Terms 6](#_Toc42494717)

[Ovid (Embase, Medline and Global Health) 6](#_Toc42494718)

[SCOPUS 6](#_Toc42494719)

[LILACS 7](#_Toc42494720)

[Risk of bias assessment 8](#_Toc42494721)

[Supplementary Table 1: Characteristics of the analysed studies (197 studies) 10](#_Toc42494722)

[Supplementary Table 2: Mean levels (mg/dl) of selected lipid biomarkers (197 studies) 30](#_Toc42494723)

[Supplementary Table 3: prevalence estimates of selected lipid biomarkers (197 studies) 37](#_Toc42494724)

[Supplementary Table 4: Selected lipid clinical guidelines from Latin America and the Caribbean 45](#_Toc42494725)

[Supplementary Table 5: Country classification into sub-regions 47](#_Toc42494726)

# PRISMA 2009 Checklist

| Section/topic | # | Checklist item | Reported on page # |
| --- | --- | --- | --- |
| TITLE | | |  |
| Title | 1 | Identify the report as a systematic review, meta-analysis, or both. | 01 |
| ABSTRACT | | |  |
| Structured summary | 2 | Provide a structured summary including, as applicable: background; objectives; data sources; study eligibility criteria, participants, and interventions; study appraisal and synthesis methods; results; limitations; conclusions and implications of key findings; systematic review registration number. | 03 |
| INTRODUCTION | | |  |
| Rationale | 3 | Describe the rationale for the review in the context of what is already known. | 04 |
| Objectives | 4 | Provide an explicit statement of questions being addressed with reference to participants, interventions, comparisons, outcomes, and study design (PICOS). | 04 |
| METHODS | | |  |
| Protocol and registration | 5 | Indicate if a review protocol exists, if and where it can be accessed (e.g., Web address), and, if available, provide registration information including registration number. | 05 |
| Eligibility criteria | 6 | Specify study characteristics (e.g., PICOS, length of follow-up) and report characteristics (e.g., years considered, language, publication status) used as criteria for eligibility, giving rationale. | 05 |
| Information sources | 7 | Describe all information sources (e.g., databases with dates of coverage, contact with study authors to identify additional studies) in the search and date last searched. | 05 |
| Search | 8 | Present full electronic search strategy for at least one database, including any limits used, such that it could be repeated. | 06 |
| Study selection | 9 | State the process for selecting studies (i.e., screening, eligibility, included in systematic review, and, if applicable, included in the meta-analysis). | 06 |
| Data collection process | 10 | Describe method of data extraction from reports (e.g., piloted forms, independently, in duplicate) and any processes for obtaining and confirming data from investigators. | 06 |
| Data items | 11 | List and define all variables for which data were sought (e.g., PICOS, funding sources) and any assumptions and simplifications made. | 07 |
| Risk of bias in individual studies | 12 | Describe methods used for assessing risk of bias of individual studies (including specification of whether this was done at the study or outcome level), and how this information is to be used in any data synthesis. | 07 |
| Summary measures | 13 | State the principal summary measures (e.g., risk ratio, difference in means). | 07 |
| Synthesis of results | 14 | Describe the methods of handling data and combining results of studies, if done, including measures of consistency (e.g., I^2^) for each meta-analysis. | 07 |
| Risk of bias across studies | 15 | Specify any assessment of risk of bias that may affect the cumulative evidence (e.g., publication bias, selective reporting within studies). | NA |
| Additional analyses | 16 | Describe methods of additional analyses (e.g., sensitivity or subgroup analyses, meta-regression), if done, indicating which were pre-specified. | NA |
| RESULTS | | |  |
| Study selection | 17 | Give numbers of studies screened, assessed for eligibility, and included in the review, with reasons for exclusions at each stage, ideally with a flow diagram. | 08 |
| Study characteristics | 18 | For each study, present characteristics for which data were extracted (e.g., study size, PICOS, follow-up period) and provide the citations. | 08 |
| Risk of bias within studies | 19 | Present data on risk of bias of each study and, if available, any outcome level assessment (see item 12). | 10 |
| Results of individual studies | 20 | For all outcomes considered (benefits or harms), present, for each study: (a) simple summary data for each intervention group (b) effect estimates and confidence intervals, ideally with a forest plot. | 08-10 |
| Synthesis of results | 21 | Present results of each meta-analysis done, including confidence intervals and measures of consistency. | 08-10 |
| Risk of bias across studies | 22 | Present results of any assessment of risk of bias across studies (see Item 15). | NA |
| Additional analysis | 23 | Give results of additional analyses, if done (e.g., sensitivity or subgroup analyses, meta-regression [see Item 16]). | NA |
| DISCUSSION | | |  |
| Summary of evidence | 24 | Summarize the main findings including the strength of evidence for each main outcome; consider their relevance to key groups (e.g., healthcare providers, users, and policy makers). | 11 |
| Limitations | 25 | Discuss limitations at study and outcome level (e.g., risk of bias), and at review-level (e.g., incomplete retrieval of identified research, reporting bias). | 12-13 |
| Conclusions | 26 | Provide a general interpretation of the results in the context of other evidence, and implications for future research. | 14 |
| FUNDING | | |  |
| Funding | 27 | Describe sources of funding for the systematic review and other support (e.g., supply of data); role of funders for the systematic review. | 02 |

# Search Terms

## **Ovid (Embase, Medline and Global Health)**

| 1 | exp Cholesterol/ |
| --- | --- |
| 2 | cholesterol.mp. |
| 3 | 1 or 2 |
|  |  |
| 4 | exp Cholesterol, LDL/ |
| 5 | ldl-cholesterol.mp. |
| 6 | low density lipoprotein cholesterol.mp. |
| 7 | 4 or 5 or 6 |
|  |  |
| 8 | exp Cholesterol, HDL/ |
| 9 | hdl-cholesterol.mp. |
| 10 | high density lipoprotein cholesterol.mp. |
| 11 | 8 or 9 or 10 |
|  |  |
| 12 | exp Triglycerides/ |
| 13 | triglycerides.mp. |
| 14 | 12 or 13 |
|  |  |
| 15 | dyslipidemi$.mp. |
| 16 | lipid disorder.mp. |
| 17 | hypercholesterolemia.mp. |
| 18 | hypertriglyceridemia.mp. |
| 19 | hyperlipidemia.mp. |
| 20 | 15 or 16 or 17 or 18 or 19 |
|  |  |
| 21 | 3 or 7 or 11 or 14 or 20 |
|  |  |
| 22 | (("Antigua and Barbuda") or ("Argentina") or ("Bahamas") or ("Barbados") or ("Belize") or ("Bolivia") or ("Brazil") or ("United States Virgin Islands") or ("British Virgin Islands") or ("Chile") or ("Colombia") or ("Costa Rica") or ("Cuba") or ("Dominica") or ("Dominican Republic") or ("Ecuador") or ("El Salvador") or ("Grenada") or ("Guatemala") or ("Guyana") or ("Haiti") or ("Honduras") or ("Jamaica") or ("Mexico") or ("Nicaragua") or ("Panama") or ("Paraguay") or ("Peru") or ("Puerto Rico") or ("Saint Kitts and Nevis") or ("Saint Lucia") or ("Saint Vincent and the Grenadines") or ("Suriname") or ("Trinidad and Tobago") or ("West Indies") or ("Uruguay") or ("Venezuela") or ("Latin America") or latin amer$ or ("South America") or south amer$ or ("Central America") or central amer$ or ("Caribbean Region")) |
|  |  |
| 23 | 21 and 22 |
| 24 | exp animals/ not humans.sh. |
| 25 | 23 not 24 |
| 26 | remove duplicates from 25 |

## **SCOPUS**

(TITLE-ABS-KEY(cholesterol) OR TITLE-ABS-KEY(LDL cholesterol) OR TITLE-ABS-KEY(low density lipoprotein cholesterol) OR TITLE-ABS-KEY(HDL cholesterol) OR TITLE-ABS-KEY(high density lipoprotein cholesterol) OR TITLE-ABS-KEY(triglycerides) OR TITLE-ABS-KEY(dyslipidemi*) OR TITLE-ABS-KEY(lipid disorder) OR TITLE-ABS-KEY(hypercholesterolemia) OR TITLE-ABS-KEY(hypertriglyceridemia) OR TITLE-ABS-KEY(hyperlipidemia)) AND (TITLE-ABS-KEY("Antigua and Barbuda") or TITLE-ABS-KEY("Argentina") or TITLE-ABS-KEY("Bahamas") or TITLE-ABS-KEY("Barbados") or TITLE-ABS-KEY("Belize") or TITLE-ABS-KEY("Bolivia") or TITLE-ABS-KEY("Brazil") or TITLE-ABS-KEY("United States Virgin Islands") or TITLE-ABS-KEY("British Virgin Islands") or TITLE-ABS-KEY("Chile") or TITLE-ABS-KEY("Colombia") or TITLE-ABS-KEY("Costa Rica") or TITLE-ABS-KEY("Cuba") or TITLE-ABS-KEY("Dominica") or TITLE-ABS-KEY("Dominican Republic") or TITLE-ABS-KEY("Ecuador") or TITLE-ABS-KEY("El Salvador") or TITLE-ABS-KEY("Grenada") or TITLE-ABS-KEY("Guatemala") or TITLE-ABS-KEY("Guyana") or TITLE-ABS-KEY("Haiti") or TITLE-ABS-KEY("Honduras") or TITLE-ABS-KEY("Jamaica") or TITLE-ABS-KEY("Mexico") or TITLE-ABS-KEY("Nicaragua") or TITLE-ABS-KEY("Panama") or TITLE-ABS-KEY("Paraguay") or TITLE-ABS-KEY("Peru") or TITLE-ABS-KEY("Puerto Rico") or TITLE-ABS-KEY("Saint Kitts and Nevis") or TITLE-ABS-KEY("Saint Lucia") or TITLE-ABS-KEY("Saint Vincent and the Grenadines") or TITLE-ABS-KEY("Suriname") or TITLE-ABS-KEY("Trinidad and Tobago") or TITLE-ABS-KEY("West Indies") or TITLE-ABS-KEY("Uruguay") or TITLE-ABS-KEY("Venezuela") or TITLE-ABS-KEY("Latin America") or TITLE-ABS-KEY(latin amer$) or TITLE-ABS-KEY("South America") or TITLE-ABS-KEY(south amer$) or TITLE-ABS-KEY("Central America") or TITLE-ABS-KEY(central amer$) or TITLE-ABS-KEY("Caribbean Region")) NOT DBCOLL(medl) AND  (LIMIT-TO ( DOCTYPE , "ar"))  AND  ( LIMIT-TO(SUBJAREA, "MEDI"))

## **LILACS**

((colesterol) OR (LDL colesterol) OR (lipoproteína de baja densidad) OR (HDL colesterol) OR (lipoproteína de alta densidad) OR (trigliceridos) OR (dislipidemias) OR (hipercolesterolemia) OR (hipertrigliceridemia) OR (hiperlipidemia)) AND(("Antigua y Barbuda") or ("Argentina") or ("Aruba") or ("Bahamas") or ("Barbados") or ("Belice") or ("Bolivia") or ("Brasil") or ("Islas Vírgenes de los Estados Unidos") or ("Islas Vírgenes Británicas") or ("Islas Caimán") or ("Chile") or ("Colombia") or ("Costa Rica") or ("Cuba") or ("Curazao") or ("Dominica") or ("Republica Dominicana") or ("Ecuador") or ("El Salvador") or ("Granada") or ("Guatemala") or ("Guyana") or ("Haití") or ("Honduras") or ("Jamaica") or ("México") or ("Nicaragua") or ("Panamá") or ("Paraguay") or ("Perú") or ("Puerto Rico") or ("San Cristóbal y Nieves ") or ("Santa Lucía") or ("San Vicente y las Granadinas ") or ("Surinam") or ("Trinidad y Tobago") or ("Turcas y Caicos ") or ("Uruguay") or ("Venezuela") or ("América Latina") or ("Latinoamérica") or ("América del Sur") or ("Sudamérica") or ("Suramérica​") or ("América Central") or ("Centroamérica") or ("América del Centro") or ("Caribe"))

# Risk of bias assessment

As described in the main document, we followed the recommendations and tool by Hoy and colleagues.^[[1]](#footnote-1)^ Below we described our rationale for grading each of the ten criteria in the risk of bias (RoB) assessment tool.

The first item in the RoB tool is: *Was the study’s target population a close representation of the national population in relation to relevant variables, e.g. age, sex, occupation?* We considered the selected studies to be “low risk of bias” because we only included population-based studies with random sampling of the general population; also, we excluded studies with one population group alone (e.g., smokers). Whether a community study is a close representation of the country, is of course arguable. However, that study would still be “more representative” than one with a selected or convenience sample. For this item the possible outcomes were “low risk of bias” and “high risk of bias”. We considered our selected studies as “low risk of bias” because even when they were not national surveys, they may still be representative or informative of the general population.

The second item in the RoB tool is: *Was the sampling frame a true or close representation of the target population?* We followed a similar logic as above. Because of the study design -population-based with random sampling- we considered that all selected studies were a true (in the case of national surveys or studies in multiple cities) or close representation (in the case of small studies conducted in a limited area) of the target population. Studies were deemed as “low risk of bias”.

The third item in the RoB tool is: *Was some form of random selection used to select the sample, OR, was a census undertaken?* As with the two first items, our inclusion criteria already prevented us from having studies with no random selection of the study population. Studies were regarded as “low risk of bias”.

The fourth item in the RoB tool is: *Was the likelihood of non-response bias minimal?* Following the recommendations by the RoB tool, some studies did not meet the threshold for “low risk of bias” with regards to the response rate. When evidence was available, reports were classified as low or high risk of bias.

The fifth item in the RoB tool is: *Were data collected directly from the subjects (as opposed to a proxy)?* All studies were deemed as “low risk of bias” in this item because of our selection criteria. We only included studies with direct measurement of any lipid biomarkers. This, by definition, implies that data were collected directly from the subjects.

The sixth item in the RoB tool is: *Was an acceptable case definition used in the study?* Half of our work was about mean levels of lipid biomarkers. As these were numeric variables, there is no case definition. The other half of our work was about prevalence estimates of dyslipidaemia traits. Here, not all studies used the same case definitions, and there could be potential explanations: i) case definitions have changed over the years, and old studies may have adhered to recommendations available at the time; ii) rather than using international recommendations, some studies used local guidelines; and iii) some studies used a different definition (e.g., percentiles) based on their research question. Regardless of these potential issues, the case definition in the original study was not “wrong”; these were just different yet accurate within their time period, geographic scope and aims. Studies were classified as “low risk of bias”. Finally, regarding our pooled prevalence estimates (see Table 1 in the main document), only consistent definitions were pooled.

The seventh item in the RoB tool is: *Was the study instrument that measured the parameter of interest (e.g .prevalence of low back pain) shown to have reliability and validity (if necessary)?* We considered studies met criteria for “low risk of bias” because they all measured lipid biomarkers, which is a reliable source of information. Information was not self-reported o gathered from other written sources.

The eighth item in the RoB tool is: *Was the same mode of data collection used for all subjects?* The methodology followed by the epidemiological studies of interest, requires a consistent protocol for all subjects. Data and blood samples collection, as well as laboratory procedures, were consistent for all subjects. We considered studies as “low risk of bias”.

The ninth item in the RoB tool is: *Was the length of the shortest prevalence period for the parameter of Interest appropriate?* We were interested in the point prevalence of dyslipidaemia traits; that is, the prevalence at the time of data collation. Studies met these criteria, thus classified as “low risk of bias”. This item would not apply for mean levels.

The tenth item in the RoB tool is: *Were the numerator(s) and denominator(s) for the parameter of interest appropriate?* For our research question, the numerator and denominator were adequate; for example, number of people with high total cholesterol over the total study population. Consequently, we thought all studies were “low risk of bias”.

Finally, the overall summary proposed by this RoB tool has three options: *i) low risk of bias when further research is very unlikely to change our confidence in the estimate; ii) moderate risk of bias when further research is likely to have an important impact on our confidence in the estimate and may change the estimate; and iii) high risk of bias when further research is very likely to have an important impact on our confidence in the estimate and is likely to change the estimate.* Because most -if not all- studies were “low risk of bias” in most items, but we cannot guarantee studies were completely free risk of bias, we deemed all studies as “moderate risk of bias”. In some cases, further research could change our confidence in the estimates, but still, we would not expect the estimates to change dramatically. This because we sought population-based studies with random sampling of the general population, and where lipid biomarkers were measured with laboratory-based methods.

A limitation of our methodology is that we assessed the RoB based on the information available in each selected report (or paper), which may not contain all details to make a comprehensive assessment of the original study. Ideally, we would have needed to investigate the original protocol, but certainly this does not happen often in any systematic review. Moreover, because of our original selection criteria, we strongly consider that the selected reports do not provide biased information to affect our results or conclusions.

# Supplementary Table 1: Characteristics of the analysed studies (197 studies)

NI: no information

| **Country** | **1st author** | **Publication year** | **Data collection year** | **Overall sample size** | **Men (%)** | **Age (mean)** | **Coverage** | **Fasting** |
| --- | --- | --- | --- | --- | --- | --- | --- | --- |
| Argentina | Vinueza^1^ | 2010 | 2004 | 1412 |  |  | Community | Yes |
| Argentina | Salazar^2^ | 2013 | 2003 | 926 | 32.83 | 51.42 | Community | Yes |
| Argentina | Rubinstein^3^ | 2015 | 2011 | 3990 |  |  | Community | Yes |
| Argentina | Salazar^4^ | 2009 | 2007 | 892 | 28.59 | 40 | Community | Yes |
| Argentina | Carbajal^5^ | 2001 |  | 1523 | 36.51 |  | Community | Yes |
| Argentina | Schneider^6^ | 2006 | 2001 | 107 | 0 |  | Community | Yes |
| Argentina | Ferrante^7^ | 2007 | 2005 | 41392 | 47.5 |  | National | NI |
| Argentina | Redruello^8^ | 2008 | 2007 | 522 | 41 | 48.7 | Community | NI |
| Argentina | Ferrante^9^ | 2011 | 2009 | 34372 |  |  | National | NI |
| Barbados | Howitt^10^ | 2015 | 2012 | 1234 | 38.09 |  | National | Yes |
| Belize | Wong-McClure^11^ | 2015 | 2005 | 1520 | 35.4 |  | National | Yes |
| Brazil | Velasquez-Melendez^12^ | 1999 | 1991 | 951 | 40.69 |  | Community | Yes |
| Brazil | Pereira^13^ | 2006 | 2000 | 1577 | 45.57 | 44.79 | Community | Yes |
| Brazil | Poletto^14^ | 1992 | 1989 | 238 | 100 |  | Community | Yes |
| Brazil | Rigo^15^ | 2008 | 2005 | 378 | 33.33 | 68.5 | Community | Yes |
| Brazil | Sesso^16^ | 2008 | 1992 | 269 | 45.7 |  | Community | NI |
| Brazil | Pimenta^17^ | 2013 | 2005 | 280 | 50.6 |  | Community | Yes |
| Brazil | Quintino-Santos^18^ | 2012 | 1996 | 1408 | 39.6 | 69 | Community | NI |
| Brazil | Togeiro^19^ | 2013 | 2007 | 1042 |  |  | Community | Yes |
| Brazil | Ota ^20^ | 2011 | 2001 | 382 | 31.68 | 79.82 | Community | Yes |
| Brazil | Pimenta^21^ | 2007 | 2001 | 287 | 48.4 |  | Community | Yes |
| Brazil | Souza^22^ | 2003 | 2001 | 1039 | 47.8 | 48.8 | Community | Yes |
| Brazil | Yokota^23^ | 2012 | 2010 | 869 | 45.1 |  | Community | Yes |
| Brazil | Petris^24^ | 2016 | 2011 | 967 | 45.5 |  | Community | NI |
| Brazil | Pirkle^25^ | 2018 | 2012 | 311 | 49.2 | 69 | Community | NI |
| Brazil | Werle^26^ | 2011 | 1994 | 187 | 36.4 | 83.6 | National | Yes |
| Brazil | Oliveira^27^ | 2012 | 2006 | 517 | 0 | 29 | Community | Yes |
| Brazil | Venturini^28^ | 2013 | 2006 | 304 | 28.6 |  | Community | NI |
| Brazil | Pavan^29^ | 1997 |  | 370 |  |  | Community | NI |
| Brazil | Roriz-Cruz^30^ | 2007 |  | 422 | 35.2 | 68.3 | Community | NI |
| Brazil | Neumann^31^ | 2007 | 2001 | 2277 | 45.7 |  | Community | Yes |
| Brazil | Nunes Filho^32^ | 2007 | 2006 | 353 | 49.3 | 40.4 | Community | Yes |
| Brazil | Gimeno^33^ | 2011 | 2006 | 930 |  | 42.42 | Community | Yes |
| Brazil | Moraes^34^ | 2012 | 2007 | 2471 | 40.3 | 47.1 | Community | Yes |
| Brazil | Garcez^35^ | 2014 | 2008 | 299 | 48.2 |  | Community | Yes |
| Brazil | Garcez^35^ | 2014 | 2008 | 263 | 47.1 |  | Community | Yes |
| Brazil | Gomes^36^ | 2013 | 2010 | 113 | 35.4 | 82.77 | Community | Yes |
| Brazil | Kaestner^37^ | 2017 | 2014 | 38069 | 40.1 | 14.6 | National | Yes |
| Brazil | Fortanelli^38^ | 2018 | 2015 | 901 | 51.6 |  | Community | Yes |
| Brazil | Marquezine^39^ | 2008 |  | 1561 | 45.48 |  | Community | Yes |
| Brazil | Mendes-Lana^40^ | 2007 |  | 185 |  | 53.82 | Community | NI |
| Brazil | Pena^41^ | 2016 | 2009 | 931 | 45.6 | 45.09 | Community | Yes |
| Brazil | Bieleman^42^ | 2014 | 1982 | 1918 |  |  | Community | No |
| Brazil | Cardoso^43^ | 2002 | 1991 | 1067 | 40.7 | 39.8 | Community | NI |
| Brazil | Buffarini^44^ | 2018 | 1993 | 917 | 47.76 | 18.5 | Community | No |
| Brazil | De Oliveira^45^ | 2010 | 1999 | 1461 | 46.4 | 44.8 | Community | Yes |
| Brazil | Martins^46^ | 1989 | 1987 | 279 |  |  | Community | Yes |
| Brazil | Fornes^47^ | 2000 | 1990 | 1328 | 41.15 |  | Community | Yes |
| Brazil | Martins^48^ | 1996 | 1991 | 1049 | 41.11 |  | Community | Yes |
| Brazil | Correa Leite^49^ | 2013 | 1997 | 1407 | 39.5 | 69.1 | Community | Yes |
| Brazil | Gus^50^ | 2002 | 2000 | 1066 | 48.2 |  | Community | Yes |
| Brazil | Marcopito^51^ | 2005 | 2001 | 2103 | 43.71 |  | Community | Yes |
| Brazil | de Oliveira^52^ | 2008 | 2006 | 1666 | 43.4 | 44 | Community | Yes |
| Brazil | de Castro^53^ | 2015 | 2009 | 462 | 38.53 | 53.9 | Community | Yes |
| Brazil | de Souza^54^ | 2016 | 2009 | 281 | 37.7 | 39.8 | Sub-national | Yes |
| Brazil | Drumond^55^ | 2011 | 2009 | 382 | 48.17 |  | Community | Yes |
| Brazil | Mendes^56^ | 2014 | 2009 | 881 | 45.74 |  | Sub-national | Yes |
| Brazil | Castro^57^ | 2016 | 2010 | 417 | 39 | 54 | Sub-national | Yes |
| Brazil | Cocate^58^ | 2013 | 2011 | 299 | 100 | 50.5 | Community | Yes |
| Brazil | Costa-Fagundes^59^ | 2018 | 2011 | 316 | 45.3 | 74.22 | Community | Yes |
| Brazil | Barbosa^60^ | 2016 | 2012 | 968 | 38 | 22.63 | Community | Yes |
| Brazil | Bernardi^61^ | 2015 | 2003 | 2063 | 48.23 | 23.9 | Community | Yes |
| Brazil | Candido^62^ | 2007 |  | 400 | 26.5 | 45.17 | Community | Yes |
| Brazil | de Oliveira^63^ | 2012 |  | 1032 | 45.77 | 43.04 | National | Yes |
| Brazil | de Oliveira^64^ | 2013 |  | 2003 | 46.43 | 42.54 | Community | Yes |
| Brazil | de Oliveira^65^ | 2017 |  | 1662 | 45.78 | 44.75 | Community | Yes |
| Brazil | Dressler^66^ | 2006 |  | 258 | 38.8 | 40.9 | Community | Yes |
| Brazil | Beleigoli^67^ | 2017 | 2000 | 1138 | 36.9 | 68 | Community | Yes |
| Brazil | Bustos^68^ | 2007 | 2002 | 2063 | 48.2 | 23.9 | Community | Yes |
| Brazil | Costa^69^ | 2010 | 2004 | 3123 |  |  | National | NI |
| Brazil | Carnelosso^70^ | 2010 | 2004 | 3275 | 39.1 | 42 | Community | NI |
| Brazil | Cabral^71^ | 2015 | 2013 | 959 | 49.2 |  | Community | Yes |
| Brazil | Matos^72^ | 2003 |  | 160 | 43.7 | 46.6 | Community | Yes |
| Chile | Jadue^73^ | 1999 | 1997 | 1669 | 26.43 |  | Community | Yes |
| Chile | Garcia Hermozo^74^ | 2017 | 2009 | 2472 | 41.4 | 46 | Sub-national | Yes |
| Chile | Vinueza^1^ | 2010 | 2004 | 1605 |  |  | Community | Yes |
| Chile | Palomo^75^ | 2007 | 2005 | 1007 | 33.7 | 45.16 | Community | Yes |
| Chile | Tejos^76^ | 2013 | 2010 | 2794 | 48.9 | 41.7 | National | Yes |
| Chile | Villanueva^77^ | 2018 | 2010 | 5248 | 40.62 | 41.57 | National | Yes |
| Chile | Rubinstein^3^ | 2015 | 2011 | 1950 |  |  | Community | Yes |
| Chile | Lanas^78^ | 2016 | 2009 | 5416 | 40.62 | 46.69 | National | NI |
| Chile | Lara^79^ | 2012 |  | 999 | 43.7 | 24.8 | Community | Yes |
| Chile | Mena^80^ | 2015 |  | 832 | 33.3 | 45 | Community | NI |
| Chile | Miquel^81^ | 1998 |  | 1991 | 40.5 | 40.9 | Community | NI |
| Chile | Mujica^82^ | 2008 |  | 1007 | 33.7 | 45.2 | Community | Yes |
| Chile | Valenzuela^83^ | 2010 | 2003 | 1883 | 44.24 |  | National | Yes |
| Chile | Group I multicentre collaborative ^84^ | 1992 | 1989 | 199 | 100 | 45.5 | Community | NI |
| Chile | Cuevas^85^ | 2008 | 1997 | 964 | 34.85 | 40.1 | Community | Yes |
| Chile | Pivatto^86^ | 2007 | 2001 | 998 | 43.7 | 24.8 | Community | Yes |
| Chile | Amigo^87^ | 2010 | 2002 | 999 | 43.74 |  | Community | Yes |
| Chile | Labrana^88^ | 2017 | 2009 | 5157 | 40.6 | 40.26 | National | Yes |
| Chile | Acevedo^89^ | 2009 | 2007 | 999 | 50.85 | 43.8 | Community | Yes |
| Chile | Acevedo^90^ | 2012 | 2005 | 1624 | 47.41 | 45 | Community | Yes |
| Chile | Berrios^91^ | 1997 | 1988 | 521 | 35.5 | 40 | Community | NI |
| Colombia | Roldan-Menco^92^ | 2017 |  | 302 | 41 | 51 | Community | Yes |
| Colombia | Pirkle^25^ | 2018 | 2012 | 374 | 49.2 | 69 | Community | NI |
| Colombia | Arbey^93^ | 2011 |  | 61 | 100 | 47.1 | Community | NI |
| Colombia | Patino-Villada^94^ | 2011 | 2009 | 357 | 39.78 |  | Community | Yes |
| Colombia | Camacho^95^ | 2018 | 2006 | 6628 | 35.9 | 50.7 | National | Yes |
| Colombia | Vinueza^1^ | 2010 | 2004 | 1511 |  |  | Community | Yes |
| Colombia | Palmett-Rios^96^ | 2017 | 2011 | 1300 | 30.6 | 40.6 | Sub-national | NI |
| Colombia | Bautista^97^ | 2006 | 2001 | 2989 | 35.76 | 35.1 | Community | No |
| Colombia | Gallo^98^ | 2013 | 2007 | 800 | 44.8 | 50.3 | Community | Yes |
| Colombia | Alayon^99^ | 2010 | 2008 | 207 | 28 | 33.5 | Community | Yes |
| Costa Rica | Campos^100^ | 1991 | 1988 | 465 | 47.74 |  | Community | Yes |
| Costa Rica | Wong-McClure^11^ | 2015 | 2005 | 1050 | 38.1 |  | Community | Yes |
| Costa Rica | Holst^101^ | 2006 | 2001 | 400 | 47 | 32.5 | Community | Yes |
| Costa Rica | Chanti-Ketterl^102^ | 2017 | 2005 | 2677 | 46 | 70.5 | National | Yes |
| Costa Rica | Campos^100^ | 1991 | 1988 | 376 | 53.72 | 40.47 | Community | Yes |
| Costa Rica | Rehkopf^103^ | 2018 | 2005 | 2827 | 47.01 |  | National | NI |
| Costa Rica | Jimenez^104^ | 1987 | 1982 | 2054 | 45.96 |  | National | Yes |
| Costa Rica | Goldman^105^ | 2011 | 2005 | 2827 | 53.7 |  | National | Yes |
| Costa Rica | Williams^106^ | 2007 | 1996 | 484 | 73.46 | 57.18 | Community | NI |
| Cuba | Salas^107^ | 2016 | 2004 | 2944 | 35 | 75.1 | Community | Yes |
| Cuba | Nordet^108^ | 2013 | 2009 | 1287 | 35.1 | 54.9 | Community | Yes |
| Dominica | Robinson^109^ | 2004 |  | 211 | 35 | 34.9 | National | NI |
| Dominican Republic | Aono^110^ | 1999 | 1993 | 1893 | 40.09 | 36.3 | National | NI |
| Dominican Republic | Salas^107^ | 2016 | 2005 | 2011 | 34.1 | 75.3 | Community | Yes |
| Dominican Republic | Dong^111^ | 2011 | 1997 | 1211 | 39 | 44.1 | Community | Yes |
| Ecuador | Encalada-Torres^112^ | 2017 | 2015 | 387 | 36.43 | 72.89 | Community | Yes |
| Ecuador | Vinueza^1^ | 2010 | 2004 | 1620 |  |  | Community | Yes |
| Ecuador | Orces^113^ | 2017 | 2010 | 2053 | 46.76 |  | National | Yes |
| Ecuador | Sisa^114^ | 2018 | 2010 | 1307 | 46.37 | 75 | National | Yes |
| El Salvador | Orantes^115^ | 2011 | 2009 | 775 | 44.26 | 39.2 | Community | Yes |
| Granada | Bansilal^116^ | 2012 | 2009 | 2827 | 42.48 |  | National | Yes |
| Guatemala | Wong-McClure^11^ | 2015 | 2005 | 904 | 26 |  | Community | Yes |
| Guatemala | Romero-Abal^117^ | 1994 |  | 107 | 26 | 69 | Community | Yes |
| Guatemala | Gregory^118^ | 2009 | 1998 | 376 | 48.14 | 24.45 | Community | Yes |
| Haiti | DeGennaro^119^ | 2018 | 2016 | 2131 | 38.9 | 40.8 | Community | NI |
| Honduras | Wong-McClure^11^ | 2015 | 2005 | 1124 | 30.3 |  | Community | Yes |
| Honduras | Hall Marti­nez^120^ | 2005 | 2003 | 246 | 42.3 | 48.21 | Community | Yes |
| Jamaica | Ferguson^121^ | 2017 | 2007 | 2231 | 30.39 | 39.4 | National | Yes |
| Jamaica | Gupta^122^ | 2010 | 1996 | 1466 | 41.47 | 45.9 | Community | Yes |
| Jamaica | Florey^123^ | 1973 |  | 696 | 33.9 |  | Community | Yes |
| Jamaica | Ferguson^124^ | 2008 | 2001 | 1972 | 33.52 | 36.3 | National | Yes |
| Jamaica | Ferguson^125^ | 2010 | 2006 | 839 | 45.05 | 18.78 | National | Yes |
| Jamaica | Tulloch-Reid^126^ | 2013 | 2008 | 1432 | 31.42 |  | National | Yes |
| Jamaica | Stringhini^127^ | 2016 | 2011 | 386 | 38.2 | 34.5 | Community | Yes |
| Mexico | Ramirez-Lopez^128^ | 2005 | 2003 | 360 |  | 8.54 | Community | Yes |
| Mexico | Vinueza^1^ | 2010 | 2004 | 1677 |  |  | Community | Yes |
| Mexico | Rosas-Saucedo^129^ | 2009 |  | 608 |  |  | Community | Yes |
| Mexico | Sanchez-Corona^130^ | 2004 |  | 163 | 41.1 | 31.7 | Community | NI |
| Mexico | Aguilar-Salinas^131^ | 2002 | 1993 | 14069 | 40.64 | 30.37 | National | Yes |
| Mexico | Aguilar-Salinas^132^ | 2011 | 2000 | 1729 |  | 36.8 | National | Yes |
| Mexico | Escobedo de la Peña^133^ | 2014 | 2004 | 1722 | 48.37 |  | Sub-national | Yes |
| Mexico | Echevarri­a-Pinto^134^ | 2006 | 2005 | 73 | 42.46 |  | Community | Yes |
| Mexico | Salas^107^ | 2016 | 2007 | 2003 | 36.69 | 74.77 | Community | Yes |
| Mexico | Rodriguez-Moran^135^ | 2001 |  | 210 | 34.76 | 40.5 | Community | Yes |
| Mexico | Rodriguez-Ramirez^136^ | 2015 |  | 4272 | 31.6 | 43.9 | Sub-national | Yes |
| Mexico | Posadas-Romero^137^ | 1995 | 1987 | 33558 | 35.88 |  | National | No |
| Mexico | Valdez^138^ | 1995 | 1991 | 1878 | 40.73 | 46.42 | Community | Yes |
| Mexico | Kumar^139^ | 2016 | 2001 | 2086 | 40.03 | 62.06 | National | Yes |
| Mexico | Hernandez^140^ | 2017 | 2012 | 194 | 22.4 | 44.2 | Community | Yes |
| Mexico | Posadas-Romero^141^ | 1994 | 1991 | 805 | 53.42 | 40.9 | Sub-national | Yes |
| Mexico | Aguilar^142^ | 1999 |  | 142 | 30.28 | 38 | Community | Yes |
| Mexico | Ferrannini^143^ | 2009 |  | 1941 | 41.58 | 47 | Community | Yes |
| Mexico | Aguilar-Salinas^144^ | 2010 | 2006 | 4040 | 46.31 | 40.3 | National | Yes |
| Mexico | Gonzales-Villalpando^145^ | 1999 |  | 2279 | 41.2 | 46.9 | Community | Yes |
| Nicaragua | Wong-McClure^11^ | 2015 | 2005 | 1587 | 37.2 |  | Community | Yes |
| Peru | Salas^107^ | 2016 | 2006 | 1933 | 38.81 | 74.77 | Community | Yes |
| Peru | Seclen^146^ | 2006 | 2000 | 612 | 31.7 |  | Community | Yes |
| Peru | Soto^147^ | 2005 | 2004 | 1000 | 24.2 |  | Community | Yes |
| Peru | Vinueza^1^ | 2010 | 2004 | 1628 |  |  | Community | Yes |
| Peru | Benzinger^148^ | 2010 | 2005 | 1448 | 47.44 | 52.4 | Community | Yes |
| Peru | Quispe^149^ | 2016 | 2011 | 3220 | 49.47 | 55.35 | Sub-national | Yes |
| Peru | Baracco^150^ | 2007 | 2002 | 271 | 36.16 | 47.37 | Community | Yes |
| Peru | Benziger^148^ | 2018 | 2010 | 3057 | 48.7 | 55.6 | Sub-national | Yes |
| Peru | Benziger^151^ | 2015 | 2010 | 3087 | 48.7 | 55.6 | Sub-national | Yes |
| Peru | Chirinos^152^ | 2014 |  | 2513 | 45.4 | 51 | Community | Yes |
| Peru | Seclen^153^○ | 1999 |  | 598 | 31.27 | 40.4 | Sub-national | Yes |
| Peru | Goldstein^154^ | 2005 | 1999 | 2237 | 52.39 | 40.6 | Sub-national | Yes |
| Peru | Medina-Lezama^155^ | 2007 | 2005 | 1878 | 46.2 | 49.09 | Community | Yes |
| Peru | Miranda^156^ | 2011 | 2007 | 1176 | 47.2 | 48 | Community | Yes |
| Peru | Malaga^157^ | 2010 | 2009 | 74 | 37.8 | 51.7 | Community | Yes |
| Peru | Gaziano^158^ | 2016 | 2010 | 3601 | 48.7 | 53.4 | Sub-national | Yes |
| Peru | Gonzales^159^ | 2013 | 2010 | 506 | 31.23 | 51.81 | Community | Yes |
| Peru | Cardenas^160^ | 2009 | 2005 | 4053 | 49.74 |  | National | Yes |
| Puerto Rico | Garcia Palmieri^161^ | 1972 |  | 5803 | 100 |  | National | Yes |
| Puerto Rico | Perez^162^ | 2011 | 2006 | 858 | 49.4 | 34.4 | Sub-national | Yes |
| Puerto Rico | Salas^107^ | 2016 | 2008 | 2009 | 32.7 | 76.3 | Community | Yes |
| Puerto Rico | Cruz-Vidal^163^ | 1979 | 1964 | 8757 | 100 | 55 | National | Yes |
| Puerto Rico | Castelli^164^ | 1977 | 1965 | 956 | 100 |  | Sub-national | Yes |
| Puerto Rico | Costas^165^ | 1978 | 1965 | 4970 | 100 |  | Community | Yes |
| Surinam | Krishnadath^166^ | 2016 | 2013 | 5748 | 48.5 | 35 | National | Yes |
| Trinidad and Tobago | Miljkovic-Gacic^167^ | 2006 |  | 202 |  | 73.1 | National | NI |
| Trinidad and Tobago | Miller^168^ | 1989 | 1979 | 2488 | 53.97 |  | Community | Yes |
| Uruguay | Rubinstein^3^ | 2015 | 2011 | 1584 |  |  | Community | Yes |
| US Virgin Islands | Tull^169^ | 2005 | 1998 | 1089 | 31.12 | 34.63 | Community | Yes |
| US Virgin Islands | Tull^170^ | 2013 | 1998 | 799 | 31.66 | 45.89 | Community | Yes |
| Venezuela | Uzcategui^171^ | 2015 | 2006 | 274 | 36.13 | 42.3 | Community | NI |
| Venezuela | Salazar^172^ | 2018 | 2008 | 2004 | 47.6 | 39.6 | Community | Yes |
| Venezuela | Bermudez^173^ | 2017 | 2014 | 1379 | 44.1 | 46.9 | Community | Yes |
| Venezuela | Fernandez^174^ | 2006 |  | 1703 | 31 | 47.02 | Community | Yes |
| Venezuela | Florez^175^ | 2005 | 2000 | 3108 | 30.44 | 43.72 | Community | Yes |
| Venezuela | Gonzales-Rivas^176^ | 2016 | 2008 | 1392 | 31.3 | 45.2 | National | Yes |
| Venezuela | Becerra^177^ | 2009 | 2007 | 109 | 32.11 | 39.1 | Community | Yes |
| Venezuela | Vinueza^1^ | 2010 | 2004 | 1824 |  |  | Community | Yes |
| Venezuela | Salas^107^ | 2016 | 2006 | 1965 | 36.5 | 72.3 | Community | Yes |
| Venezuela | Bermudez^178^ | 2013 | 2013 | 1807 | 44.7 | 39.2 | Community | Yes |

1. Vinueza R, Boissonnet CP, Acevedo M, et al. Dyslipidemia in seven Latin American cities: CARMELA study. *Prev Med (Baltim)*. 2010;50(3):106-111. internal-pdf://89.119.235.165/Vinueza-2010-Dyslipidemia in seven Latin Ameri.pdf.

2. Salazar MR, Carbajal HA, Espeche WG, et al. Identifying cardiovascular disease risk and outcome: Use of the plasma triglyceride/high-density lipoprotein cholesterol concentration ratio versus metabolic syndrome criteria. *J Intern Med*. 2013;273(6):595-601. internal-pdf://0390127940/Salazar-2013-Identifying cardiovascular diseas.pdf.

3. Rubinstein AL, Irazola VE, relli M, et al. Multiple cardiometabolic risk factors in the Southern Cone of Latin America: a population-based study in Argentina, Chile, and Uruguay. *Int J Cardiol*. 2015;183:82-88. internal-pdf://99.124.224.12/Rubinstein-2015-Multiple cardiometabolic risk.pdf.

4. Salazar MR, Carbajal HA, Marillet AG, et al. Glomerular filtration rate, cardiovascular risk factors and insulin resistance. *Med (B Aires)*. 2009;69(5):541-546. internal-pdf://247.242.225.146/Glomerular filtration rate, cardiovascular ris.pdf.

5. Carbajal H, Salazar M, Riondet B, et al. Variables asociadas a hipertensión arterial en una region de la Argentina. *Med [BAires]*. 2001;61:801-809. file:///C:/Users/Usuario/Downloads/base de datos/150. ARG_XXXX_Carbajal.pdf.

6. Schneider RJ, Barengo N, Haapala I, Tavella M. Association between dietary habits, education, serum triglycerides and blood cholesterol among women of Cabildo, Buenos Aires. *Med (B Aires)*. 2006;66(6):517-525. internal-pdf://181.31.226.0/Association between dietary habits, education.pdf.

7. Ferrante D, Virgolini M. Encuesta Nacional de Factores de Riesgo 2005: resultados principales: prevalencia de factores de riesgo de enfermedades cardiovasculares en la Argentina. *Rev argent cardiol*. 2007:20-29. file:///C:/Users/Usuario/Downloads/base de datos/45. ARG_2005_Ferrante.pdf.

8. Redruello MF, Calderón G, Masoli O, et al. Prevalencia de factores de riesgo y riesgo cardiovascular global en la población de Tres Lomas. *Rev Argent Cardiol*. 2008;76(6):450-458. internal-pdf://0630578493/Prevalencia de factores de riesgo y riesgo car.pdf.

9. Ferrante D, Linetzky B, Konfino J, King A, Virgolini M, Laspiur S. ENCUESTA NACIONAL DE FACTORES DE RIESGO 2009: EVOLUCIÓN DE LA EPIDEMIA DE ENFERMEDADES CRÓNICAS NO TRANSMISIBLES EN ARGENTINA. ESTUDIO DE CORTE TRANSVERSAL. *Rev Argent Salud Pública*. 2011;2(6):34-41. file:///C:/Users/Usuario/Downloads/base de datos/46. ARG_2009_Ferrante.pdf.

10. Howitt C, Hambleton IR, Rose AMC, et al. Social distribution of diabetes, hypertension and related risk factors in Barbados: a cross-sectional study. *BMJ Open*. 2015;5(12):e008869. http://ovidsp.ovid.com/ovidweb.cgi?T=JS&PAGE=reference&D=med8&NEWS=N&AN=26685026 https://www.ncbi.nlm.nih.gov/pmc/articles/PMC4691788/pdf/bmjopen-2015-008869.pdf.

11. Wong-McClure RA, Gregg EW, Barceló A, et al. Prevalence of metabolic syndrome in Central America: a cross-sectional population-based study. *Rev Panam Salud Publica*. 2015;38(3):202-208. internal-pdf://84.162.236.112/Wong-McClure-2015-Prevalence of metabolic synd.pdf.

12. Velásquez-Meléndez G, Martins IS, Cervato AM, Fornés NS, De Marucci FNM, Coelho LT. Relationship between stature, overweight and central obesity in the adult population in Sao Paulo, Brazil. *Int J Obes*. 1999;23(6):639-644. internal-pdf://0074246686/Relationship between stature, overweight and c.pdf.

13. Pereira AC, Sposito AC, Mota GF, et al. Endothelial nitric oxide synthase gene variant modulates the relationship between serum cholesterol levels and blood pressure in the general population: new evidence for a direct effect of lipids in arterial blood pressure. *Atherosclerosis*. 2006;184(1):193-200. internal-pdf://0873519429/Pereira-2006-Endothelial nitric oxide synthase.pdf.

14. Poletto L, Pezzotto S, Morini J. Blood lipid associations in 18 year-old men. *Rev Saude Publica*. 1992;26(5):316-320. internal-pdf://0428071503/Blood lipid associations in 18 year-old men.pdf.

15. Rigo JC, Vieira JL, Dalacorte RR, Reichert CL. Prevalence of metabolic syndrome in an elderly community: comparison between three diagnostic methods. *Arq Bras Cardiol*. 2009;93(2):85-91. internal-pdf://0974249511/Prevalence of metabolic syndrome in an elderly.pdf.

16. Sesso R, Prado F, Vicioso B, Ramos LR. Prospective study of progression of kidney dysfunction in community-dwelling older adults. *Nephrology (Carlton)*. 2008;13(2):99-103. internal-pdf://235.149.227.41/Sesso-2008-Prospective study of progression of.pdf.

17. Pimenta AM, Felisbino-Mendes MS, Velasquez-Melendez G. Clustering and combining pattern of metabolic syndrome components in a rural Brazilian adult population. *Sao Paulo Med J*. 2013;131(4):213-219. internal-pdf://69.50.218.1/Pimenta-2013-Clustering and combining pattern.pdf.

18. Quintino-Santos S, Regina ra, Lima-Costa MF, et al. Homozygosity for the APOE E4 allele is solely associated with lower cognitive performance in Brazilian community-dwelling older adults: the Bambui Study. *Rev Bras Psiquiatr*. 2012;34(4):440-445. internal-pdf://226.181.220.36/Quintino-Santos-2012-Homozygosity for the APOE.pdf.

19. Togeiro SM, Carneiro G, Ribeiro Filho F, et al. Consequences of obstructive sleep apnea on metabolic profile: a Population-Based Survey. *Obesity (Silver Spring)*. 2013;21(4):847-851. internal-pdf://150.191.230.54/Togeiro-2013-Consequences of obstructive sleep.pdf.

20. Ota VK, Chen ES, Ejchel TF, et al. APOA4 polymorphism as a risk factor for unfavorable lipid serum profile and depression: a cross-sectional study. *J Investig Med*. 2011;59(6):966-970. internal-pdf://73.166.214.41/APOA4 polymorphism as a risk factor for unfavo.pdf.

21. Pimenta AM, Kac G, Gazzinelli A, Corrêa-Oliveira R, Velásquez-Meléndez G. Associação entre obesidade central, triglicerídeos e hipertensão arterial em uma área rural do Brasil. *Arq Bras Cardiol*. 2008;90(6):419-425. internal-pdf://208.167.218.17/Associação entre obesidade central, triglicerí.pdf.

22. Souza LJ de, Souto Filho JTD, Souza TF de, et al. Prevalence of dyslipidemia and risk factors in Campos dos Goytacazes, in the Brazilian State of Rio de Janeiro. *Arq Bras Cardiol*. 2003;81(3):257-264. doi:10.1590/s0066-782x2003001100005

23. Yokota RT de C, Iser BPM, Andrade RLM, et al. Vigilância de fatores de risco e proteção para doenças e agravos não transmissíveis em município de pequeno porte, Brasil, 2010. *Epidemiol serv saúde*. 2012;21(1):55-68. internal-pdf://71.31.237.59/Vigilância de fatores de risco e proteção para.pdf.

24. Petris AJ, Souza RKT de, Bortoletto MSS. [Public sector participation in the supply of dyslipidemia medication in a population-based study]. *Particip do Set publico no fornecimento Medicam para dislipidemias em Estud base Popul*. 2016;21(12):3899-3906. internal-pdf://68.22.217.241/Petris-2016-[Public sector participation in th.pdf.

25. Pirkle CM, Wu YY, Zunzunegui M-V, Gomez JF. Model-based recursive partitioning to identify risk clusters for metabolic syndrome and its components: findings from the International Mobility in Aging Study. *BMJ Open*. 2018;8(3):e018680. internal-pdf://183.95.218.32/Pirkle-2018-Model-based recursive partitioning.pdf.

26. Werle MH, Moriguchi E, Fuchs S, et al. Risk factors for cardiovascular disease in the very elderly: results of a cohort study in a city in southern Brazil. *Eur J Cardiovasc Prev Rehabil*. 2011;18(3):369-377. internal-pdf://223.89.235.243/Risk factors for cardiovascular disease in the.pdf.

27. Oliveira BFA de, Mourão D de S, Gomes N, et al. Prevalência de hipertensão arterial em comunidades ribeirinhas do Rio Madeira, Amazônia Ocidental Brasileira. *Cad Saude Publica*. 2013;29(8):1617-1630. internal-pdf://107.119.212.240/Oliveira-2013-Prevalência de hipertensão arter.pdf.

28. Venturini CD, Engroff P, Gomes I, De Carli GA. Prevalência de obesidade associada à ingestão calórica, glicemia e perfil lipídico em uma amostra populacional de idosos do Sul do Brasil. *Rev bras geriatr gerontol*. 2013;16(3):591-601. internal-pdf://6.163.131.30/Prevalência de obesidade associada à ingestão.pdf.

29. Pavan L, Casiglia E, Pauletto P, et al. Blood pressure, serum cholesterol and nutritional state in Tanzania and in the Amazon: comparison with an Italian population. *J Hypertens*. 1997;15(10):1083-1090. internal-pdf://83.164.215.253/Blood pressure, serum cholesterol and nutritio.pdf.

30. Roriz-Cruz M, Rosset I, Wada T, et al. Cognitive impairment and frontal-subcortical geriatric syndrome are associated with metabolic syndrome in a stroke-free population. *Neurobiol Aging*. 2007;28(11):1723-1736. internal-pdf://0670818238/Roriz-Cruz-2007-Cognitive impairment and front.pdf.

31. Neumann AICP, Martins IS, Marcopito LF, Araujo EAC. [Dietary patterns associated with risk factors for cardiovascular disease in a Brazilian city]. *Padroes Aliment Assoc a fatores risco para doencas Cardiovasc entre Resid um Munic Bras*. 2007;22(5):329-339. http://ovidsp.ovid.com/ovidweb.cgi?T=JS&PAGE=reference&D=med5&NEWS=N&AN=18198042.

32. Nunes Filho JR, Debastiani D, Nunes A, Daros ra, Peres KG. Prevalência de Fatores de risco cardiovascular em adultos de Luzerna, Santa Catarina, 2006. *Arq Bras Cardiol*. 2007;89(5):319-324. internal-pdf://0591254487/Prevalência de Fatores de risco cardiovascular.pdf.

33. Gimeno SGA, Mondini L, Moraes SA de, Freitas ICM de. Padrões de consumo de alimentos e fatores associados em adultos de Ribeirão Preto, São Paulo, Brasil: Projeto OBEDIARP. *Cad Saude Publica*. 2011;27(3):533-545. http://www.scielo.br/scielo.php?script=sci_arttext&pid=S0102-311X2011000300013 http://www.scielo.br/pdf/csp/v27n3/13.pdf.

34. Moraes SA de, Checchio MV, Freitas ICM de. Dislipidemia e fatores associados em adultos residentes em Ribeirão Preto, SP: resultados do Projeto EPIDCV. *Arq Bras Endocrinol Metab*. 2013;57(9):691-701. http://www.scielo.br/scielo.php?script=sci_arttext&pid=S0004-27302013000900004 http://www.scielo.br/pdf/abem/v57n9/a04v57n9.pdf.

35. Garcez MR, Pereira JL, de Mello Fontanelli M, Marchioni DML, Fisberg RM. Prevalence of dyslipidemia according to the nutritional status in a representative sample of São Paulo. *Arq Bras Cardiol*. 2014;103(6):476-484. https://www.scopus.com/inward/record.uri?eid=2-s2.0-84923261698&doi=10.5935%2Fabc.20140156&partnerID=40&md5=4767ec36b6a4fcff752e772c75b1e2aa https://www.ncbi.nlm.nih.gov/pmc/articles/PMC4290738/pdf/abc-103-06-0476.pdf.

36. Gomes IC, Santos VR, Christofaro DGD, Santos LL, Freitas Junior IF. The most frequent cardiovascular risk factors in Brazilian aged 80 years or older. *J Appl Gerontol*. 2013;32(4):408-421. http://ovidsp.ovid.com/ovidweb.cgi?T=JS&PAGE=reference&D=med7&NEWS=N&AN=25474682.

37. Kaestner TL, Bento VF, Pazin DC, et al. Prevalence of high cholesterol levels suggestive of familial hypercholesterolemia in Brazilian adolescents: Data from the study of cardiovascular risk in adolescents. *J Clin Lipidol*. 2018;12(2):403-408. http://ovidsp.ovid.com/ovidweb.cgi?T=JS&PAGE=reference&D=prem&NEWS=N&AN=29429893 https://ac.els-cdn.com/S1933287417305445/1-s2.0-S1933287417305445-main.pdf?_tid=adb15a01-f923-4e81-9860-b1c94e39ee86&acdnat=1551036759_d7402dc6921c927c4150b654fa933f51.

38. Fontanelli M de M, Nogueira LR, Garcez MR, et al. [Validity of self-reported high cholesterol in the city of Sao Paulo, Brazil, and factors associated with this information’s sensitivity]. *Validade da autorreferencia Colest Elev na Cid Sao Paulo, Bras e fatores Assoc a Sensib dessa Inf*. 2018;34(12):e00034718. http://ovidsp.ovid.com/ovidweb.cgi?T=JS&PAGE=reference&D=prem&NEWS=N&AN=30517313 http://www.scielo.br/pdf/csp/v34n12/1678-4464-csp-34-12-e00034718.pdf.

39. Marquezine GF, Oliveira CM, Pereira AC, Krieger JE, Mill JG. Metabolic syndrome determinants in an urban population from Brazil: Social class and gender-specific interaction. *Int J Cardiol*. 2008;129(2):259-265. https://www.scopus.com/inward/record.uri?eid=2-s2.0-50349097935&doi=10.1016%2Fj.ijcard.2007.07.097&partnerID=40&md5=6ead8904463dc2ac8a304dfe0d55c292 https://ac.els-cdn.com/S0167527307016543/1-s2.0-S0167527307016543-main.pdf?_tid=ffedde13-9c38-45b4-b26a-3e.

40. Mendes-Lana A, Pena GG, Freitas SN, et al. Apolipoprotein E polymorphism in Brazilian dyslipidemic individuals: Ouro Preto study. *Brazilian J Med Biol Res = Rev Bras Pesqui medicas e Biol*. 2007;40(1):49-56. http://ovidsp.ovid.com/ovidweb.cgi?T=JS&PAGE=reference&D=med5&NEWS=N&AN=17224996 http://www.scielo.br/pdf/bjmbr/v40n1/6491.pdf.

41. Pena GG, Martinez-Perez A, Dutra MS, et al. Genetic determinants of cardiometabolic risk factors in rural families in Brazil. *Am J Hum Biol*. 2016;28(5):619-626. internal-pdf://197.85.216.216/Pena-2016-Genetic determinants of cardiometabo.pdf.

42. Bielemann RM, Ramires VV, Gigante DP, Hallal PC, Horta BL. Longitudinal and cross-sectional associations of physical activity with triglyceride and HDLc levels in young male adults. *J Phys Act Heal*. 2014;11(4):784-789. doi:10.1123/jpah.2012-0175

43. Cardoso E, Salas Martins I, Fornari L, C.Monachini M, De Pádua Mansur A, Caramelli B. ALTERAÇÕES ELETROCARDIOGRÁFICAS E SUA RELAÇÃO COM OS FATORES DE RISCO PARA DOENÇA ISQUÊMICA DO CORAÇÃO EM POPULAÇÃO DA ÁREA METROPOLITANA DE SÃO PAULO. *Rev Assoc Med Bras*. 2002;1(12):8. file:///C:/Users/Usuario/Downloads/base de datos/44. BRA_1991_Cardoso.pdf.

44. Buffarini R, Restrepo-Méndez MC, Silveira VM, et al. Growth across life course and cardiovascular risk markers in 18-year-old adolescents: The 1993 Pelotas birth cohort. *BMJ Open*. 2018;8(1):1-8. doi:10.1136/bmjopen-2017-019164

45. Alvim RO, Freitas SR, Ferreira NE, et al. APOE polymorphism is associated with lipid profile, but not with arterial stiffness in the general population. *Lipids Health Dis*. 2010;9(1):128. doi:10.1186/1476-511X-9-128

46. Martins IS, Coelho LT, Matos IM, Mazzilli RN, Trigo M, Wilson D. [Dyslipidemia and some risk factors in the population outside of the metropolitan region of Sao Paulo, SP-Brazil. A pilot study]. *Dislipidemias e alguns fatores risco Assoc em uma Popul periferica da Reg Metrop Sao Paulo, SP-Brasil Um Estud Pilot*. 1989;23(3):236-243. http://ovidsp.ovid.com/ovidweb.cgi?T=JS&PAGE=reference&D=med3&NEWS=N&AN=2617118.

47. Fornés NS, Martins IS, Hernan M, Velásquez-Meléndez G, Ascherio A. Food frequency consumption and lipoproteins serum levels in the population of an urban area, Brazil. *Rev Saude Publica*. 2000;34(4):380-387. http://www.scielo.br/scielo.php?script=sci_arttext&pid=S0034-89102000000400011&lng=en&nrm=iso http://www.scielo.br/pdf/rsp/v34n4/2536.pdf.

48. Martins IS, Nunes Marucci MDF, Cervato AM, Okani ET, Mazzilli RN, Casajus MI. Atherosclerotic cardiovascular disease, lipemic disorders, hypertension, obesity and diabetes millitus in the population of a metropolitan area of southeastern Brazil. II - Lipemic disorders. *Rev Saude Publica*. 1996;30(1):75-84. https://www.scopus.com/inward/record.uri?eid=2-s2.0-0030072895&partnerID=40&md5=7eb4d180374604fcdebf15eceff114f9.

49. Léa Correa Leite M, Fernanda Lima-Costa M, Moriguchi EH. Age-related trends of blood pressure levels by apolipoprotein E genotype: the Bambuì cohort study of ageing (1997–2008). *Hypertens Res*. 2013;36(3):270-276. doi:10.1038/hr.2012.175

50. Gus I, Fischmann A, Medina C. Prevalence of risk factors for coronary artery disease in the Brazilian State of Rio Grande do Sul. *Arq Bras Cardiol*. 2002;78(5):478-490. http://ovidsp.ovid.com/ovidweb.cgi?T=JS&PAGE=reference&D=med4&NEWS=N&AN=12045846 http://www.scielo.br/pdf/abc/v78n5/9378.pdf.

51. Marcopito LF, Rodrigues SSF, Pacheco MA, Shirassu MM, Goldfeder AJ, Moraes MA de. [Prevalence of a set of risk factors for chronic diseases in the city of Sao Paulo, Brazil]. *Prevalencia alguns fatores risco para doencas cronicas na Cid Sao Paulo*. 2005;39(5):738-745. http://ovidsp.ovid.com/ovidweb.cgi?T=JS&PAGE=reference&D=med5&NEWS=N&AN=16254649.

52. de Oliveira CM, Pereira AC, de Andrade M, Soler JM, Krieger JE. Heritability of cardiovascular risk factors in a Brazilian population: Baependi Heart Study. *BMC Med Genet*. 2008;9. doi:10.1186/1471-2350-9-32

53. de Castro MA, Baltar VT, Marchioni DML, Fisberg RM. Sex differences in serum leptin and its relation to markers of cardiometabolic risk in middle-aged adults: Evidence from a population-based study. *Nutrition*. 2015;31(3):491-497. doi:10.1016/j.nut.2014.09.007

54. de Souza WN, Norde MM, Oki É, et al. Association between 25-hydroxyvitamin D and inflammatory biomarker levels in a cross-sectional population-based study, São Paulo, Brazil. *Nutr Res*. 2016;36(1):1-8. doi:10.1016/j.nutres.2015.10.006

55. Drumond MG, Da E, Metabólica S. Estabilidade da síndrome metabólica e dos seus componentes em duas comunidades rurais de Minas Gerais. 2011. file:///C:/Users/Usuario/Downloads/base de datos/48. BRA_2009_Drumond.PDF.

56. Felisbino-Mendes MS, Jansen AK, Gomes CS, Velásquez-Meléndez G. Avaliação dos fatores de risco cardiovasculares em uma população rural Brasileira. *Cad Saude Publica*. 2014;30(6):1183-1194. doi:10.1590/0102-311X00103213

57. Castro MA, Baltar VT, Marchioni DM, Fisberg RM. Examining associations between dietary patterns and metabolic CVD risk factors: A novel use of structural equation modelling. *Br J Nutr*. 2016;115(9):1586-1597. doi:10.1017/S0007114516000556

58. Cocate PG, de Oliveira A, Hermsdorff HHM, et al. Benefits and relationship of steps walked per day to cardiometabolic risk factor in Brazilian middle-aged men. *J Sci Med Sport*. 2014;17(3):283-287. doi:10.1016/j.jsams.2013.04.017

59. Fagundes LC, Fernandes MH, Brito TA, Coqueiro R da S, Carneiro JAO. Prevalência e fatores associados à cintura hipertrigliceridêmica em idosos: Um estudo de base populacional. *Cienc e Saude Coletiva*. 2018;23(2):607-616. doi:10.1590/1413-81232018232.02862016

60. Barbosa JB, dos Santos AM, Barbosa MM, et al. Síndrome metabólica, resistência insulínica e outros fatores de risco cardiovascular em universitários. *Cienc e Saude Coletiva*. 2016;21(4):1123-1136. doi:10.1590/1413-81232015214.10472015

61. Bernardi JR, Pinheiro TV, Mueller NT, et al. Cesarean delivery and metabolic risk factors in young adults: A Brazilian birth cohort study. *Am J Clin Nutr*. 2015;102(2):295-301. doi:10.3945/ajcn.114.105205

62. Cândido APC, Ferreira S, Lima AA, et al. Lipoprotein(a) as a risk factor associated with ischemic heart disease: Ouro Preto Study. *Atherosclerosis*. 2007;191(2):454-459. doi:10.1016/j.atherosclerosis.2006.04.031

63. Alvim RDO, Santos PCJL, Nascimento RM, et al. BDKRB2 +9/-9 polymorphism is associated with higher risk for diabetes mellitus in the Brazilian general population. *Exp Diabetes Res*. 2012;2012. doi:10.1155/2012/480251

64. De Oliveira Alvim R, Santos PCJL, Musso MM, et al. Impact of diabetes mellitus on arterial stiffness in a representative sample of an urban Brazilian population. *Diabetol Metab Syndr*. 2013;5(1):1-8. doi:10.1186/1758-5996-5-45

65. Alvim R de O, Mourao-Junior CA, Magalhães GL, et al. Non-hdl cholesterol is a good predictor of the risk of increased arterial stiffness in postmenopausal women in an urban brazilian population. *Clinics*. 2017;72(2):106-110. doi:10.6061/clinics/2017(02)07

66. Dressler WW, Balieiro MC, Ribeiro RP, Dos Santos JE. Depressive symptoms and C-reactive protein in a Brazilian urban community. *Brazilian J Med Biol Res*. 2006;39(8):1013-1019. doi:10.1590/S0100-879X2006000800003

67. Beleigoli AM, Diniz MDFH, Boersma E, Silva JL, Lima-Costa MF, Ribeiro AL. The effects of weight and waist change on the risk of long-term mortality in older adults- The Bambuí (Brazil) Cohort Study of Aging. *J Nutr Heal Aging*. 2017;21(8):861-866. doi:10.1007/s12603-016-0858-z

68. Bustos P, da Silva AAM, Amigo H, Bettiol H, Barbieri MA. Metabolic syndrome in young adults from two socioeconomic Latin American settings. *Nutr Metab Cardiovasc Dis*. 2007;17(8):581-589. doi:10.1016/j.numecd.2006.06.001

69. Costa LC. Prevalência de fatores de risco para doenças não transmissíveis na população adulta de 18 capitais brasileiras: um inquérito domiciliar. 2010:83-83.

70. Carnelosso ML, Barbosa MA, Porto CC, e Silva SA, de Carvalho MM, Oliveira ALI. Prevalência de fatores de risco para doenças cardiovasculares na região leste de Goiânia (GO). *Cienc e Saude Coletiva*. 2010;15(SUPPL. 1):1073-1080. doi:10.1590/s1413-81232010000700014

71. Cabral da Rocha AL, Pereira PF, Pessoa MC, et al. Fenotipo cintura hipertrigliceridémica y alteraciones cardiometabólicas en adultos Brasileños. *Nutr Hosp*. 2015;32(3):1099-1106. doi:10.3305/nh.2015.32.3.9305

72. Matos AC, Ladeia AM. Assessment of cardiovascular risk factors in a rural community in the Brazilian state of Bahia. *Arq Bras Cardiol*. 2003;81(3):291-302. http://ovidsp.ovid.com/ovidweb.cgi?T=JS&PAGE=reference&D=med4&NEWS=N&AN=14569373 http://www.scielo.br/pdf/abc/v81n3/17334.pdf.

73. Jadue Hund L, Vega Morales J, Escobar S MC, et al. Factores de riesgo para las enfermedades no transmisibles: metodología y resultados globales de la encuesta de base del programa CARMEN: conjunto de acciones para la reducción multifactorial de las enfermedades no transmisibles. *Rev Med Chil*. 1999;127(8):1004-1013. http://www.scielo.cl/scielo.php?script=sci_arttext&pid=S0034-98871999000800017&lng=es&nrm=iso&tlng=es.

74. Garcia-Hermoso A, Hackney AC, Ramirez-Velez R. Ideal cardiovascular health predicts lower risk of abnormal liver enzymes levels in the Chilean National Health Survey (2009-2010). *PLoS One*. 2017;12(10):e0185908. http://ovidsp.ovid.com/ovidweb.cgi?T=JS&PAGE=reference&D=medc&NEWS=N&AN=29049384 https://www.ncbi.nlm.nih.gov/pmc/articles/PMC5648121/pdf/pone.0185908.pdf.

75. Palomo G I, Icaza N G, Mujica E V, et al. Prevalencia de factores de riesgo cardiovascular clásicos en población adulta de Talca, Chile, 2005. *Rev Med Chil*. 2007;135(7):904-912. internal-pdf://160.24.215.19/Palomo G-2007-Prevalencia de factores de riesg.pdf.

76. Tejos R, Padilla O, Pizarro M, et al. [Serum levels of alanine aminotransferase in Chilean population: analysis of results of the national health survey 2009-2010]. *Niveles sericos alanino-aminotransferasa en Poblac Chil Anal los Result la encuesta Nac salud 2009-2010*. 2013;141(7):909-916. internal-pdf://124.97.229.107/Tejos-2013-[Serum levels of alanine aminotrans.pdf.

77. Villanueva B, Arteaga A, Maiz A, Cortes VA. Abdominal obesity is a common finding in normal and overweight subjects of Chile and is associated with increased frequency of cardiometabolic risk factors. *PLoS One*. 2018;13(3):e0194644. internal-pdf://76.159.235.71/Villanueva-2018-Abdominal obesity is a common.pdf.

78. Lanas F, Serón P, Muñoz S, Margozzini P, Puig T. Latin American Clinical Epidemiology Network Series – Paper 7: Central obesity measurements better identified risk factors for coronary heart disease risk in the Chilean National Health Survey (2009–2010). *J Clin Epidemiol*. 2017;86:111-116. https://www.scopus.com/inward/record.uri?eid=2-s2.0-85021970039&doi=10.1016%2Fj.jclinepi.2016.04.018&partnerID=40&md5=f5be3dee47b240b47b92ac53f952a462 https://ac.els-cdn.com/S089543561630556X/1-s2.0-S089543561630556X-main.pdf?_tid=098d8b20-a4fb-4f61-bc1f-.

79. Lara M, Bustos P, Amigo H, Silva C, Rona RJ. Is waist circumference a better predictor of blood pressure, insulin resistance and blood lipids than body mass index in young Chilean adults? *BMC Public Health*. 2012;12(1). https://www.scopus.com/inward/record.uri?eid=2-s2.0-84864828598&doi=10.1186%2F1471-2458-12-638&partnerID=40&md5=f85b83410473d83ec837a16579cb3955.

80. Mena C, Fuentes E, Ormazabal Y, Palomo-Velez G, Palomo I. Role of access to parks and markets with anthropometric measurements, biological markers, and a healthy lifestyle. *Int J Environ Health Res*. 2015;25(4):373-383. http://ovidsp.ovid.com/ovidweb.cgi?T=JS&PAGE=reference&D=med8&NEWS=N&AN=25236622 https://www.tandfonline.com/doi/pdf/10.1080/09603123.2014.958134?needAccess=true.

81. Miquel JF, Covarrubias C, Villaroel L, et al. Genetic epidemiology of cholesterol cholelithiasis among Chilean Hispanics, Amerindians, and Maoris. *Gastroenterology*. 1998;115(4):937-946. http://ovidsp.ovid.com/ovidweb.cgi?T=JS&PAGE=reference&D=med4&NEWS=N&AN=9753497.

82. Mujica V, Leiva E, Icaza G, et al. Evaluation of metabolic syndrome in adults of Talca city, Chile. *Nutr J*. 2008;7:14. http://ovidsp.ovid.com/ovidweb.cgi?T=JS&PAGE=reference&D=med6&NEWS=N&AN=18482457 https://www.ncbi.nlm.nih.gov/pmc/articles/PMC2397433/pdf/1475-2891-7-14.pdf.

83. Valenzuela AA, Maiz A, Margozzini P, et al. [Prevalence of metabolic syndrome among Chilean adults]. *Prevalencia Sindr Metab en Poblac adulta Chil Datos la Encuesta Nac Salud 2003*. 2010;138(6):707-714. internal-pdf://156.153.233.161/Valenzuela.pdf.

84. Group I multicentre collaborative. Risk factors for cardiovascular disease in the developing world. A multicentre collaborative study in the International clinical epidemiology network (INCLEN). *J Clin Epidemiol*. 1992;45(8):841-847. doi:10.1016/0895-4356(92)90067-W

85. Cuevas A, Molina A, Rigotti A, et al. Trends in obesity and diabetes prevalence in a Chilean urban population: 1993-2001. *Metab Syndr Relat Disord*. 2008;6(3):219-222. doi:10.1089/met.2008.0018

86. Pivatto I, Bustos P, Amigo H, Acosta AM, Arteaga A. Association between proinsulin, insulin, proinsulin/insulin ratio, and insulin resistance status with the metabolic syndrome. *Arq Bras Endocrinol Metab*. 2007;51(7):1128-1133. internal-pdf://76.196.218.63/Pivatto-2007-Association between proinsulin, i.pdf.

87. Amigo H, Bustos P, Alvarado ME, et al. Size at birth and lipoprotein concentrations in adulthood: Two prospective studies in Latin American cities. *J Epidemiol Community Health*. 2010;64(10):855-859. doi:10.1136/jech.2008.078345

88. Labrana AM, Duran E, Martinez MA, et al. [Effects of a lower body weight or waist circumference on cardiovascular risk]. *Menor peso corporal, indice masa Corpor y perimetro cintura se Asoc a una disminucion en factores riesgo Cardiovasc en Poblac Chil Find from Chil Heal Surv*. 2017;145(5):585-594. http://ovidsp.ovid.com/ovidweb.cgi?T=JS&PAGE=reference&D=medl&NEWS=N&AN=28898334.

89. Acevedo M, Arnaíz P, Corbalán R, et al. Modificación del grosor intima-media carotídeo según factores de riesgo clásicos y síndrome metabólico con o sin inflamación. *Rev Chil Cardiol*. 2009;28(4):337-348. doi:10.4067/s0718-85602009000300001

90. Acevedo M, Krämer V, Tagle R, et al. Relación colesterol total a HDL y colesterol no HDL: Los mejores indicadores lipídicos de aumento de grosor de la íntima media carotidea. *Rev Med Chil*. 2012;140(8):969-976. doi:10.4067/S0034-98872012000800001

91. Berrios X, Koponen T, Huiguang T, Khaltaev N, Puska P, Nissinen A. Distribution and prevalence of major risk factors of noncommunicable diseases in selected countries: The WHO Inter-Health Programme. *Bull World Health Organ*. 1997;75(2):99-108. file:///C:/Users/Usuario/Downloads/base de datos/39. CHI_1988_Berrios.pdf.

92. Roldan-Menco C, Diaz-Perez A, Barrios-Puerta Z, Pinto-Aragon EE. [Metabolic syndrome and concentrations of uric acid and ultrasensitive C-reactive protein]. *Conc acido urico y proteina c React ultrasensible con el Sindr Metab*. 2017;19(5):603-608. internal-pdf://238.108.223.96/Roldan-Menco-2017-[Metabolic syndrome and conc.pdf.

93. Arbey Mesa J, Fabián Suárez M, Arbeláez A, et al. Falta de relación entre el nivel de actividad física con marcadoresde riesgo cardiovascular y síndrome metabólico en hombresaparentemente sanos. *Endocrinol y Nutr*. 2011;58(2):68-74. doi:10.1016/j.endonu.2011.01.004

94. Patiño-Villada FA, Arango-Vélez EF, Quintero-Velásquez MA, Arenas-Sosa MM. Factores de riesgo cardiovascular en una población urbana de Colombia. *Rev Salud Publica*. 2011;13(3):433-445. internal-pdf://216.205.215.160/Patiño-Villada-2011-Factores de riesgo cardiov.pdf.

95. Camacho PA, Otero J, Pérez M, et al. The spectrum of the dyslipidemia in Colombia: The PURE study. *Int J Cardiol*. 2019;284:111-117. doi:10.1016/j.ijcard.2018.10.090

96. Palmett Ríos HE. Estudio transversal sobre estilos de vida saludable y su relación con el colesterol HDL en la población adulta. *Rev colomb cardiol*. 2017;24(5):523-531. internal-pdf://220.137.214.181/Estudio transversal sobre estilos de vida salu.pdf.

97. Bautista LE, Orãstegui M, Vera LM, Prada GE, Orozco LC, Herrã¡n OF. Prevalence and impact of cardiovascular risk factors in Bucaramanga, Colombia: Results from the Countrywide Integrated Noncommunicable Disease Intervention Programme (CINDI/CARMEN) baseline survey. *Eur J Prev Cardiol*. 2006;13(5):769-775. doi:10.1097/01.hjr.0000219113.40662.dd

98. Gallo JA, Ochoa JE, Kepa Balparda J, Aristizábal D. Puntos de corte del perímetro de la cintura para identificar sujetos con resistencia a la insulina en una población colombiana. *Acta Med Colomb*. 2013;38(3):118-126. http://www.scielo.org.co/scielo.php?script=sci_arttext&pid=S0120-24482013000300006.

99. Alayón AN, Ariza S, Baena K, Lambis L, Martínez L, Benítez L. Búsqueda activa y evaluación de factores de riesgo cardiovascular en adultos jóvenes, Cartagena de Indias, 2007. *Biomedica*. 2010;30(2):238-244. doi:10.7705/biomedica.v30i2.186

100. Campos H, Bailey SM, Gussak LS, Siles X, Ordovas JM, Schaefer EJ. Relations of body habitus, fitness level, and cardiovascular risk factors including lipoproteins and apolipoproteins in a rural and urban Costa Rican population. *Arterioscler Thromb*. 1991;11(4):1077-1088. doi:10.1161/01.atv.11.4.1077

101. Holst Schumancher I, Monge Rojas R, Barrantes Santamaría M. Niveles séricos de homocisteína total y lipoproteínas en adultos jóvenes de áreas rurales y urbanas de Costa Rica. *Arch latinoam nutr*. 2006;56(4):335-341. http://www.scielo.org.ve/scielo.php?script=sci_arttext&pid=S0004-06222006000400004&lng=es&nrm=iso&tlng=es.

102. Chanti-Ketterl M, Gamaldo A, Andel R, Thorpe RJ. The Association Between Lipoproteins, Disability, and Physical Function Among Older Costa Rican Adults. *J Aging Health*. 2018;30(5):758-777. doi:10.1177/0898264317690866

103. Rehkopf DH, Duong A, Dow WH, Rosero-Bixby L. Life-course BMI and biomarkers in persons aged 60 years or older: a comparison of the USA and Costa Rica. *Public Health Nutr*. 2018:1-10. internal-pdf://122.178.221.155/Life-course BMI and biomarkers in persons aged.pdf.

104. Jiménez M JG, Castro A V, Piza L J, Díaz W G, Valverde P, Díaz A C. Colesterol y triglicéridos en la población costarricense: interpretación de los resultados obtenidos en la Encuesta Nacional de Nutrición de 1982. *Rev Costarric Cienc Med*. 1987;8(2):89-95.

105. Goldman N, Turra CM, Rosero-Bixby L, Weir D, Crimmins E. Do biological measures mediate the relationship between education and health: A comparative study. *Soc Sci Med*. 2011;72(2):307-315. http://ovidsp.ovid.com/ovidweb.cgi?T=JS&PAGE=reference&D=medc&NEWS=N&AN=21159415.

106. Williams ES, Baylin A, Campos H. Adipose tissue arachidonic acid and the metabolic syndrome in Costa Rican adults. *Clin Nutr*. 2007;26(4):474-482. internal-pdf://188.127.236.2/Williams-2007-Adipose tissue arachidonic acid.pdf.

107. Salas A, Acosta D, Ferri CP, et al. The prevalence, correlates, detection and control of diabetes among older people in low and middle income countries. A 10/66 dementia research group population-based survey. *PLoS One*. 2016;11(2). internal-pdf://0197910618/The prevalence, correlates, detection and cont.PDF.

108. Nordet P, Mendis S, Duenas A, et al. Total cardiovascular risk assessment and management using two prediction tools, with and without blood cholesterol. *MEDICC Rev*. 2013;15(4):36-40. internal-pdf://0892588952/Total cardiovascular risk assessment and manag.pdf.

109. Robinson MT, Wilson TW, Nicholson GA, et al. AGT and RH blood group polymorphisms affect blood pressure and lipids in Afro-Caribbeans. *J Hum Hypertens*. 2004;18(5):351-363. internal-pdf://201.176.222.102/Robinson-2004-AGT and RH blood group polymorph.pdf.

110. Aono H. Lipoprotein(a) Concentrations in Healthy Subjects in the Dominican Republic. 1999:64-70. file:///C:/Users/Usuario/Downloads/base de datos/71. REP_1993_Aono.pdf.

111. Dong C, Beecham A, Wang L, et al. Genetic loci for blood lipid levels identified by linkage and association analyses in Caribbean Hispanics. *J Lipid Res*. 2011;52(7):1411-1419. doi:10.1194/jlr.P013672

112. Encalada-torres L, Macero R, Tenecela E, Toledo C, Wong S. Transaminasas séricas y síndrome metabólico en adultos mayores de 65 años de la sierra ecuatoriana. *Acta Bioquímica Clínica Latinoam*. 2017;51(4):603-608. file:///C:/Users/Usuario/Downloads/base de datos/146. ECU_2015_Encalada.pdf.

113. Orces CH, Montalvan M, Tettamanti D. Prevalence of abdominal obesity and its association with cardio metabolic risk factors among older adults in Ecuador. *Diabetes Metab Syndr*. 2017;11:S727-S733. internal-pdf://78.220.213.250/Prevalence of abdominal obesity and its associ.pdf.

114. Sisa I. Gender differences in cardiovascular risk assessment in elderly adults in Ecuador: Evidence from a national survey. *J Investig Med*. 2018. internal-pdf://90.12.228.50/sisa2018.pdf.

115. Orantes CM, Herrera R, Almaguer M, et al. Chronic kidney disease and associated risk factors in the Bajo Lempa region of El Salvador: Nefrolempa study, 2009. *MEDICC Rev*. 2011;13(4):14-22. internal-pdf://223.255.213.93/Chronic kidney disease and associated risk fac.pdf.

116. Bansilal S, Vedanthan R, Woodward M, et al. Cardiovascular risk surveillance to develop a nationwide health promotion strategy: The grenada heart project. *Glob Heart*. 2012;7(2):87-94. doi:10.1016/j.gheart.2012.06.002

117. Romero Abal ME, Mendoza I, Ramírez I de, et al. Relación entre lípidos plasmáticos y vitaminas liposolubles en anciano peri-urbanos de Guatemala. *Arch latinoam nutr*. 1994;44(3):140-144. internal-pdf://105.206.223.174/art-3.pdf.

118. Gregory CO, Martorell R, Venkat Narayan KM, Ramirez-Zea M, Stein AD. Five-year changes in adiposity and cardio-metabolic risk factors among Guatemalan young adults. *Public Health Nutr*. 2009;12(2):228-235. https://www.scopus.com/inward/record.uri?eid=2-s2.0-62249171316&doi=10.1017%2FS1368980008003443&partnerID=40&md5=e173976f219854ab4236cabe09d47fef https://www.cambridge.org/core/services/aop-cambridge-core/content/view/D53B27616A5A12B4C096EAED55A38BAD/S136.

119. Degennaro V, Malcolm S, Crompton L, et al. Community-based diagnosis of non-communicable diseases and their risk factors in rural and urban Haiti: A cross-sectional prevalence study. *BMJ Open*. 2018;8(4):1-8. doi:10.1136/bmjopen-2017-020317

120. Hall Martínez J, Hall Reyes J, Alvarenga Thiebaud M, Gómez O. Prevalencia de hipertensión arterial en adultos de El Progreso. *Rev Med Hondur*. 2005;73(2):60-64.

121. Ferguson TS, Younger-Coleman NOM, Tulloch-Reid MK, et al. Educational Health Disparities in Cardiovascular Disease Risk Factors: Findings from Jamaica Health and Lifestyle Survey 2007–2008. *Front Cardiovasc Med*. 2017;4(May):1-11. doi:10.3389/fcvm.2017.00028

122. Gupta R, Ejebe K, Butler J, et al. Association of common DNA sequence variants at 33 genetic loci with blood lipids in individuals of African ancestry from Jamaica. *Hum Genet*. 2010;128(5):557-561. http://ovidsp.ovid.com/ovidweb.cgi?T=JS&PAGE=reference&D=med6&NEWS=N&AN=20839009 https://link.springer.com/content/pdf/10.1007%2Fs00439-010-0887-3.pdf.

123. Florey C du V, McDonald H, McDonald J, Miall WE. The prevalence of diabetes in a rural population of Jamaican adults. *Int J Epidemiol*. 1972;1(2):157-166. http://ovidsp.ovid.com/ovidweb.cgi?T=JS&PAGE=reference&D=med1&NEWS=N&AN=4669190.

124. Ferguson TS, Younger NOM, Tulloch-Reid MK, et al. Prevalence of prehypertension and its relationship to risk factors for cardiovascular disease in Jamaica: Analysis from a cross-sectional survey. *BMC Cardiovasc Disord*. 2008;8:1-9. doi:10.1186/1471-2261-8-20

125. Ferguson TS, Tulloch-Reid MK, Younger NOM, et al. Prevalence of the metabolic syndrome and its components in relation to socioeconomic status among Jamaican young adults: A cross-sectional study. *BMC Public Health*. 2010;10. doi:10.1186/1471-2458-10-307

126. Tulloch-Reid MK, Younger NO, Ferguson TS, et al. Excess Cardiovascular Risk Burden in Jamaican Women Does Not Influence Predicted 10-Year CVD Risk Profiles of Jamaica Adults: An Analysis of the 2007/08 Jamaica Health and Lifestyle Survey. *PLoS One*. 2013;8(6):e66625. internal-pdf://145.3.230.210/Tulloch-Reid-2013-Excess Cardiovascular Risk B.pdf.

127. Stringhini S, Forrester TE, Plange-Rhule J, et al. The social patterning of risk factors for noncommunicable diseases in five countries: evidence from the modeling the epidemiologic transition study (METS). *BMC Public Health*. 2016;16:956. internal-pdf://0638182685/Stringhini-2016-The social patterning of risk.pdf.

128. Ramírez-López E, Grijalva-Haro MI, Valencia ME, Ponce JA, Artalejo E. Effect of a School Breakfast Program on the prevalence of obesity and cardiovascular risk factors in children. *Salud Publica Mex*. 2005;47(2):126-133. internal-pdf://125.53.220.239/Effect of a School Breakfast Program on the pr.pdf.

129. Rosas-Saucedo J, San Roman-Torres S, Eguia A, Rosas-Guzman J. Prevalence of metabolic syndrome in the city of Celaya, Mexico. *Can J Diabetes*. 2009;33(3):309. internal-pdf://160.253.223.205/Prevalence of metabolic syndrome in the city o.pdf.

130. Sanchez-Corona J, Flores-Martinez SE, Machorro-Lazo M V, et al. Polymorphisms in candidate genes for type 2 diabetes mellitus in a Mexican population with metabolic syndrome findings. *Diabetes Res Clin Pract*. 2004;63(1):47-55. internal-pdf://87.85.225.209/Sanchez-Corona-2004-Polymorphisms in candidate.pdf.

131. Aguilar-Salinas CA, Rojas R, Gómez-Pérez FJ. Prevalence and Characteristics of Early-Onset Type 2 Diabetes in Mexico. *Am J Med*. 2002;113(02):569-574. file:///C:/Users/Usuario/Downloads/base de datos/98. MEX_1993_Aguilar.pdf.

132. Aguilar-Salinas CA, Canizales-Quinteros S, Rojas-Martínez R, et al. The non-synonymous Arg230Cys variant (R230C) of the ATP-binding cassette transporter A1 is associated with low HDL cholesterol concentrations in Mexican adults: A population based nation wide study. *Atherosclerosis*. 2011;216(1):146-150. doi:10.1016/j.atherosclerosis.2010.10.049

133. Escobedo-de la Peña J, de Jesús-Pérez R, Schargrodsky H, Champagne B. Prevalencia de dislipidemias en la ciudad de México y su asociación con otros factores de riesgo cardiovascular. Resultados del estudio CARMELA. *Gac Med Mex*. 2014;150(2):128-136. file:///C:/Users/Usuario/Downloads/base de datos/105. MEX_2004_Escobedo.pdf.

134. Echavarría-Pinto M, Hernández-Lomelí A. Síndrome metabólico en adultos de 20 a 40 años en comunidad rural mexicana. *Rev Med Inst Mex Seguro*. 2006;44(4):329-335. file:///C:/Users/Usuario/Downloads/base de datos/79. MEX_2005_Echavarria.pdf.

135. Rodriguez-Moran M, Guerrero-Romero F. The parental phenotype of diabetes, but not of essential hypertension, is linked to the development of metabolic syndrome in Mexican individuals. *Acta Diabetol*. 2001;38(2):87-91. internal-pdf://150.251.222.149/The parental phenotype of diabetes, but not of.pdf.

136. Rodriguez-Ramirez M, Simental-Mendia LE, Gonzalez-Ortiz M, et al. Prevalence of Prehypertension in Mexico and Its Association With Hypomagnesemia. *Am J Hypertens*. 2015;28(8):1024-1030. internal-pdf://157.173.223.18/Rodriguez-Ramir-2015-Prevalence of Prehyperten.pdf.

137. Posadas-Romero C, Tapia-Conyer R, Lerman-Garber I, et al. Cholesterol levels and prevalence of hypercholesterolemia in a Mexican adult population. *Atherosclerosis*. 1995;118(2):275-284. internal-pdf://209.143.218.110/Posadas-Romero-1995-Cholesterol levels and pre.pdf.

138. Valdez R, o C, Mitchell BD, Haffner SM, Stern MP. Differential impact of obesity in related populations. *Obes Res*. 1995;3:223s-232s. internal-pdf://206.213.233.130/Differential impact of obesity in related popu.pdf.

139. Kumar A, Wong R, Ottenbacher KJ, Al Snih S. Prediabetes, undiagnosed diabetes, and diabetes among Mexican adults: findings from the Mexican Health and Aging Study. *Ann Epidemiol*. 2016;26(3):163-170. http://ovidsp.ovid.com/ovidweb.cgi?T=JS&PAGE=reference&D=med8&NEWS=N&AN=26872919 https://ac.els-cdn.com/S1047279716300035/1-s2.0-S1047279716300035-main.pdf?_tid=5202f681-c193-476e-8f61-fbe8fbf1791c&acdnat=1551036791_2b369406c735573fbd20e5c980e98017.

140. Hern, ez ME, Morales-Romero J, et al. Association of Urinary Activity of MMP-2 with Microalbuminuria in an Isolated Sample of Subjects Living in High Altitude Rural Locations in Mexico. *High Alt Med Biol*. 2017;18(3):209-218. http://ovidsp.ovid.com/ovidweb.cgi?T=JS&PAGE=reference&D=medl&NEWS=N&AN=28459597.

141. Posadas-Romero C, Yamamoto-Kimura L, Lerman-Garber I, et al. The prevalence of NIDDM and associated coronary risk factors in Mexico City. *Diabetes Care*. 1994;17(12):1441-1448. internal-pdf://134.106.218.188/posadas-romero1994.pdf.

142. Aguilar CA, Talavera G, Ordovas JM, et al. The apolipoprotein E4 allele is not associated with an abnormal lipid profile in a Native American population following its traditional lifestyle. *Atherosclerosis*. 1999;142(2):409-414. doi:10.1016/S0021-9150(98)00251-2

143. Ferrannini E, Massari M, Nannipieri M, Natali A, Lopez Ridaura R, Gonzales-Villalpando C. Plasma glucose levels as predictors of diabetes: The Mexico City diabetes study. *Diabetologia*. 2009;52(5):818-824. doi:10.1007/s00125-009-1289-8

144. Aguilar-Salinas CA, Gómez-Pérez FJ, Rull J, Villalpando S, Barquera S, Rojas R. Prevalence of dyslipidemias in the Mexican National Health and Nutrition Survey 2006. *Salud Publica Mex*. 2010;52(2):S44-S53. doi:10.1590/s0036-36342010000700008

145. González-Villalp, o C, Stern MP, et al. Prevalence of hypertension in a Mexican population according to the Sixth Report of the Joint National Committee on Prevention, Detection, Evaluation and Treatment of High Blood Pressure. *J Cardiovasc Risk*. 1999;6(3):177-181. https://www.scopus.com/inward/record.uri?eid=2-s2.0-0032774498&partnerID=40&md5=0d945bb862c7a6e4d8685c7521279921.

146. Seclen S, Villena A, Larrad MT, et al. Prevalence of the metabolic syndrome in the mestizo population of peru. *Metab Syndr Relat Disord*. 2006;4(1):1-6. internal-pdf://107.13.226.47/Seclen-2006-Prevalence of the metabolic syndro.pdf.

147. Soto C V, Vergara W E, Neciosup P E. Prevalencia y factores de riesgo de sindrome metabolico en poblacion adulta del Departamento de Lambayeque, Peru - 2004. *Rev Peru Med Exp Salud Publica*. 2005;22(4):254-261. internal-pdf://0623109295/Prevalencia y factores de riesgo de sindrome m.pdf.

148. Benziger CP, Zavala-Loayza JA, Bernabe-Ortiz A, et al. Low prevalence of ideal cardiovascular health in Peru. *Heart*. 2018;104(15):1251-1256. doi:10.1136/heartjnl-2017-312255

149. Quispe R, Benziger CP, Bazo-Alvarez JC, et al. The Relationship between Socioeconomic Status and CV Risk Factors: The CRONICAS Cohort Study of Peruvian Adults. *Glob Heart*. 2016;11(1):121-130. internal-pdf://0774560882/Quispe-2016-The Relationship between Socioecon.pdf.

150. Baracco R, Mohanna S. A Comparison of the Prevalence of Metabolic Syndrome and Its Components in High and Low Altitude Populations in Peru. *Metab Syndr Relat Disord*. 2007;5(1). doi:10.1089/met.2005.0019

151. Benziger CP, Bernabé-Ortiz A, Gilman RH, et al. Metabolic abnormalities are common among South American hispanics subjects with normal weight or excess body weight: The CRONICAS cohort study. *PLoS One*. 2015;10(11):1-15. doi:10.1371/journal.pone.0138968

152. Chirinos DA, Medina-Lezama J, Arguelles W, et al. Metabolic syndrome as an underlying disease entity and its relationship to subclinical atherosclerosis in Andean hispanics. *Metab Syndr Relat Disord*. 2014;12(1):49-55. doi:10.1089/met.2013.0092

153. Seclen Santistebán S, Leey Casella J, Villena Pacheco AE, et al. Prevalencia de Obesidad, Diabetes Mellitus, Hipertensión Arterial e Hipocolesterolemia como Factores de Riesgo Coronario y Cerebrovascular en Población Adulta de la Costa, Sierra y Selva del Perú. *Acta méd peru*. 1999;17(1):8-12. http://sisbib.unmsm.edu.pe/BVRevistas/acta_medica/1999_n1/prevalencia.htm.

154. Goldstein J, Jacoby E, del Aguila R, Lopez A. Poverty is a predictor of non-communicable disease among adults in Peruvian cities. *Prev Med (Baltim)*. 2005;41(3):800-806. http://ovidsp.ovid.com/ovidweb.cgi?T=JS&PAGE=reference&D=med5&NEWS=N&AN=16061280.

155. Medina-Lezama J, Zea-Diaz H, Morey-Vargas OL, et al. Prevalence of the metabolic syndrome in Peruvian Andean hispanics: the PREVENCION study. *Diabetes Res Clin Pract*. 2007;78(2):270-281. http://ovidsp.ovid.com/ovidweb.cgi?T=JS&PAGE=reference&D=med5&NEWS=N&AN=17524517 https://ac.els-cdn.com/S016882270700277X/1-s2.0-S016882270700277X-main.pdf?_tid=14de8f2d-970e-426d-ab7f-41ff3826c357&acdnat=1551036968_97490a73d1ab7b33ca91ced34b06870c.

156. Jaime MJ, Gilman RH, Smeeth L. Differences in cardiovascular risk factors in rural, urban and rural-to-urban migrants in Peru. *Heart*. 2011;97(10):787-796. http://ovidsp.ovid.com/ovidweb.cgi?T=JS&PAGE=reference&D=medc&NEWS=N&AN=21478383.

157. Málaga G, Zevallos-Palacios C, Lazo M de los Á, Huayanay C. High frequency of dyslipidemia and impaired fasting glycemia in a high altitude Peruvian population. *Rev Peru Med Exp Salud Publica*. 2010;27(4):557-561. http://www.ncbi.nlm.nih.gov/pubmed/21308195. Accessed March 30, 2018.

158. Gaziano TA, Abrahams-Gessel S, Alam S, et al. Comparison of Nonblood-Based and Blood-Based Total CV Risk Scores in Global Populations. *Glob Heart*. 2016;11(1):37-46.e2. http://ovidsp.ovid.com/ovidweb.cgi?T=JS&PAGE=reference&D=med8&NEWS=N&AN=27102021 http://spiral.imperial.ac.uk/bitstream/10044/1/54291/6/Comparison of Nonblood.pdf.

159. Gonzales GF, Tapia V. [Association of high altitude-induced hypoxemia to lipid profile and glycemia in men and women living at 4,100m in the Peruvian Central Andes]. *Asoc los Difer niveles hipoxemia en la altura con el Perf lipidico y la glucemia en varones y mujeres a 4100m altitud en los Andes Cent del Peru*. 2013;60(2):79-86. http://ovidsp.ovid.com/ovidweb.cgi?T=JS&PAGE=reference&D=med7&NEWS=N&AN=22925953.

160. Quintana HC, Abanto JS, Arbieto LR, Tasayco FM. Prevalencia del síndrome metabólico en personas a partir de 20 años de edad. Perú, 2005. *Rev Esp Salud Publica*. 2009;83(2):257-265. doi:10.1590/s1135-57272009000200009

161. Garcia Palmieri M, Costas Jr R, Schiffman J. Interrelationship of serum lipids with relative weight, blood glucose, and physical activity. *Circulation*. 1972;45(4):829-836. http://ovidsp.ovid.com/ovidweb.cgi?T=JS&PAGE=reference&D=emcl1&NEWS=N&AN=292076861.

162. Perez CM, Ortiz AP, Guzman M, Suarez E. Distribution and correlates of the metabolic syndrome in adults living in the San Juan Metropolitan Area of Puerto Rico. *P R Health Sci J*. 2012;31(3):114-122. internal-pdf://176.227.217.132/Perez-2012-Distribution and correlates of the.pdf.

163. Cruz-Vidal M, Costas R, Garcia-Palmieri MR, Sorlie PD, Hertzmark E. Factors related to diabetes mellitus in Puerto Rican men. *Diabetes*. 1979;28(4):300-307. doi:10.2337/diab.28.4.300

164. Castelli WP, Cooper GR, Doyle JT, et al. Distribution of triglyceride and total, LDL and HDL cholesterol in several populations: A cooperative lipoprotein phenotyping study. *J Chronic Dis*. 1977;30(3):147-169. doi:10.1016/0021-9681(77)90082-0

165. Costas R, Garcia-Palmieri MR, Nazario E, Sorlie PD. Relation of lipids, weight and physical activity to incidence of coronary heart disease: The puerto rico heart study. *Am J Cardiol*. 1978;42(4):653-658. doi:10.1016/0002-9149(78)90637-9

166. Krishnadath ISK, Nahar-van Venrooij LM, Jaddoe VW V, Toelsie JR. Ethnic differences in prediabetes and diabetes in the Suriname Health Study. *BMJ open diabetes Res care*. 2016;4(1):e000186. http://ovidsp.ovid.com/ovidweb.cgi?T=JS&PAGE=reference&D=prem&NEWS=N&AN=27403324.

167. Miljkovic-Gacic I, Bunker CH, Ferrell RE, et al. Lipoprotein subclass and particle size differences in Afro-Caribbeans, African Americans, and white Americans: associations with hepatic lipase gene variation. *Metabolism*. 2006;55(1):96-102. http://ovidsp.ovid.com/ovidweb.cgi?T=JS&PAGE=reference&D=med5&NEWS=N&AN=16324926 https://ac.els-cdn.com/S002604950500301X/1-s2.0-S002604950500301X-main.pdf?_tid=3a1608e4-d3bd-4f61-a3cf-604b8fbe9bec&acdnat=1551036994_79283844aad16cb79ba08b7dda0d6cda.

168. Miller GJ, Beckles GL, Maude GH, et al. Ethnicity and other characteristics predictive of coronary heart disease in a developing community: principal results of the St James Survey, Trinidad. *Int J Epidemiol*. 1989;18(4):808-817. http://ovidsp.ovid.com/ovidweb.cgi?T=JS&PAGE=reference&D=med3&NEWS=N&AN=2621016.

169. Tull ES, Laporte RE. Metabolic syndrome among Caribbean-born persons living in the U.S. Virgin Islands. *Rev Panam Salud Publica*. 2005;18(6):418-426. internal-pdf://0487646805/Metabolic syndrome among Caribbean-born person.pdf.

170. Tull ES. Assessment of the ability of the triglyceride to high density lipoprotein cholesterol ratio to discriminate insulin resistance among Caribbean-born black persons with and without Hispanic ethnicity. *West Indian Med J*. 2013;62(2):109-113. http://ovidsp.ovid.com/ovidweb.cgi?T=JS&PAGE=reference&D=med7&NEWS=N&AN=24564058.

171. Uzcategui E, Valery L, Uzcategui L, Gomez Perez R, Marquina D, Baptista T. Prevalence of the metabolic syndrome, insulin resistance index, leptin and thyroid hormone levels in the general population of Merida (Venezuela). *Invest Clin*. 2015;56(2):169-181. internal-pdf://0.97.12.139/10.1.1.1028.569.pdf.

172. Salazar J, Bermudez V, Calvo M, et al. Optimal cutoff for the evaluation of insulin resistance through triglyceride-glucose index: A cross-sectional study in a Venezuelan population. *F1000Research*. 2017;6:1337. internal-pdf://110.41.224.199/Salazar-2017-Optimal cutoff for the evaluation.pdf.

173. Bermúdez V, Salazar J, Graterol M, Rojas J. Importance of high triglycerides levels between novel coronary risk factors. *Rev Colomb Cardiol*. 2017;24(6). file:///C:/Users/Usuario/Downloads/base de datos/65. VEN_2014_Bermúdez.pdf.

174. Fernández V, Clavell E, J.Villasmil J. Niveles basales de insulina en una población del estado Zulia , Venezuela. *Invest clin*. 2014;47(June 2006):167-177. file:///C:/Users/Usuario/Downloads/base de datos/85. VEN_XXXX_Fernández.pdf.

175. Florez H, Silva E, ez V, et al. Prevalence and risk factors associated with the metabolic syndrome and dyslipidemia in White, Black, Amerindian and Mixed Hispanics in Zulia State, Venezuela. *Diabetes Res Clin Pract*. 2005;69(1):63-77. http://ovidsp.ovid.com/ovidweb.cgi?T=JS&PAGE=reference&D=med5&NEWS=N&AN=15955388 https://ac.els-cdn.com/S0168822705000070/1-s2.0-S0168822705000070-main.pdf?_tid=b6b34263-81bb-4485-94fc-23e37b7cfb62&acdnat=1551036480_a73a35a62a0cbbd499366355d682156d.

176. Gonzalez-Rivas JP, Jose Garcia Santiago R, Ugel E, Brajkovich I, Risquez A, Nieto-Martinez R. High prevalence and poor control of hypertension in five venezuelan populations: the VEMSOLS study. *Invest Clin*. 2016;57(4):364-376. http://ovidsp.ovid.com/ovidweb.cgi?T=JS&PAGE=reference&D=medl&NEWS=N&AN=29938985.

177. Becerra Leal AV, Valery TL, Del P, et al. POBLACIÓN URBANA DE MUCUCHÍES , MÉRIDA. *Rev Venez Endocrinol y Metab*. 2009;7(3):16-22. file:///C:/Users/Usuario/Downloads/base de datos/120. VEN_2007_Becerra.pdf.

178. Bermúdez V, Rojas J, Salazar J, et al. Variations of lipoprotein(a) levels in the metabolic syndrome: A report from the Maracaibo City metabolic syndrome prevalence study. *J Diabetes Res*. 2013;2013. doi:10.1155/2013/416451

# Supplementary Table 2: Mean levels (mg/dl) of selected lipid biomarkers (197 studies)

| **Country** | **1st author** | **Publication year** | **Data collection year** | **Total cholesterol** | **LDL-cholesterol** | **HDL-cholesterol** | **Triglycerides** |
| --- | --- | --- | --- | --- | --- | --- | --- |
| Argentina | Vinueza | 2010 | 2004 | 201 | 126.1 | 52.5 | 114.3 |
| Argentina | Salazar | 2013 | 2003 | 231.63 | 142.69 | 58.39 | 140.83 |
| Argentina | Rubinstein | 2015 | 2011 | 199.99 | 125.25 | 45.25 | 155.39 |
| Argentina | Salazar | 2009 | 2007 |  |  | 61.7 | 124.72 |
| Argentina | Carbajal | 2001 |  |  |  |  |  |
| Argentina | Schneider | 2006 | 2001 | 209 |  |  | 124 |
| Argentina | Ferrante | 2007 | 2005 |  |  |  |  |
| Argentina | Redruello | 2008 | 2007 | 196 | 126 | 43.3 | 138 |
| Argentina | Ferrante | 2011 | 2009 |  |  |  |  |
| Barbados | Howitt | 2015 | 2012 |  |  |  |  |
| Belize | Wong-McClure | 2015 | 2005 |  |  |  |  |
| Brazil | Velasquez-Melendez | 1999 | 1991 | 184.75 | 44.7 | 117.04 | 113.87 |
| Brazil | Pereira | 2006 | 2000 | 214.4 | 142.21 | 45.36 | 137.61 |
| Brazil | Poletto | 1992 | 1989 | 174.7 | 121.5 | 52.8 |  |
| Brazil | Rigo | 2008 | 2005 | 204 | 129.67 | 43.33 | 130 |
| Brazil | Sesso | 2008 | 1992 | 202 | 129 | 39 | 147 |
| Brazil | Pimenta | 2013 | 2005 |  |  |  |  |
| Brazil | Quintino-Santos | 2012 | 1996 |  |  | 49.2 | 151.1 |
| Brazil | Togeiro | 2013 | 2007 | 190.52 | 111.53 | 53.41 | 128.09 |
| Brazil | Ota | 2011 | 2001 | 215.9 | 131.85 | 54.36 | 149.51 |
| Brazil | Pimenta | 2007 | 2001 |  |  |  |  |
| Brazil | Souza | 2003 | 2001 | 187.65 | 104.35 | 48.4 | 150.08 |
| Brazil | Yokota | 2012 | 2010 |  |  |  |  |
| Brazil | Petris | 2016 | 2011 |  |  |  |  |
| Brazil | Pirkle | 2018 | 2012 |  |  | 53.33 | 139.7 |
| Brazil | Werle | 2011 | 1994 | 211.6 | 139.1 | 45.5 | 137.2 |
| Brazil | Oliveira | 2012 | 2006 | 163 |  |  | 148.54 |
| Brazil | Venturini | 2013 | 2006 | 209.95 | 132.7 | 45.68 | 172.07 |
| Brazil | Pavan | 1997 |  | 185.8 |  |  |  |
| Brazil | Roriz-Cruz | 2007 |  | 188.77 | 117.64 | 43.35 | 138.72 |
| Brazil | Neumann | 2007 | 2001 |  |  |  |  |
| Brazil | Nunes Filho | 2007 | 2006 |  |  |  |  |
| Brazil | Gimeno | 2011 | 2006 |  |  |  |  |
| Brazil | Moraes | 2012 | 2007 |  |  |  |  |
| Brazil | Garcez | 2014 | 2008 | 187 | 112 | 48 | 139 |
| Brazil | Garcez | 2014 | 2008 | 206 | 126 | 52 | 141 |
| Brazil | Igor Conterato Gomes | 2013 | 2010 | 200.91 | 110.23 |  |  |
| Brazil | Fortanelli | 2018 | 2015 |  |  |  |  |
| Brazil | Marquezine | 2008 |  |  |  |  |  |
| Brazil | Mendes-Lana | 2007 |  | 200.21 | 102.08 | 67.01 | 151.3 |
| Brazil | Pena | 2016 | 2009 | 195.7 |  | 48.55 | 105.67 |
| Brazil | Bieleman | 2014 | 1982 |  |  | 51.6 | 97.5 |
| Brazil | Cardoso | 2002 | 1991 |  |  |  |  |
| Brazil | Buffarini | 2018 | 1993 | 162.27 | 90.65 | 56.29 | 72.89 |
| Brazil | de Oliveira | 2010 | 1999 | 214.4 | 142.49 | 45.3 | 137.06 |
| Brazil | Martins | 1989 | 1987 |  |  |  |  |
| Brazil | Fornes | 2000 | 1990 |  |  |  |  |
| Brazil | Martins | 1996 | 1991 | 186.08 | 117.1 | 44.97 | 116.49 |
| Brazil | Correa Leite | 2013 | 1997 |  | 155 | 49.2 | 151.2 |
| Brazil | Gus | 2002 | 2000 |  |  |  |  |
| Brazil | Marcopito | 2005 | 2001 |  |  |  |  |
| Brazil | de Oliveira | 2008 | 2006 | 180.78 | 98.66 | 55.9 | 133.3 |
| Brazil | de Castro | 2015 | 2009 | 197.9 | 120.6 | 50 | 138.4 |
| Brazil | de Souza | 2016 | 2009 | 182.12 | 109.28 | 47.42 | 109.57 |
| Brazil | Drumond | 2011 | 2009 |  |  |  |  |
| Brazil | Mendes | 2014 | 2009 |  |  |  |  |
| Brazil | Castro | 2016 | 2010 |  |  |  |  |
| Brazil | Cocate | 2013 | 2011 | 211.29 |  | 44.47 | 118.87 |
| Brazil | Costa-Fagundes | 2018 | 2011 |  |  |  |  |
| Brazil | Barbosa | 2016 | 2012 |  |  | 44.04 | 85.78 |
| Brazil | Bernardi | 2015 | 2003 | 167.9 | 96.1 | 48.3 | 82.58 |
| Brazil | Candido | 2007 |  | 31.18 | 30.92 | 31.18 | 31.02 |
| Brazil | de Oliveira | 2012 |  | 192.27 |  |  | 152.76 |
| Brazil | de Oliveira | 2013 |  | 199.6 | 128.8 | 46.8 | 122.3 |
| Brazil | de Oliveira | 2017 |  | 214.33 | 142.13 | 45.39 | 139.05 |
| Brazil | Dressler | 2006 |  | 187.2 | 112.6 |  |  |
| Brazil | Beleigoli | 2017 | 2000 | 230 |  |  |  |
| Brazil | Bustos | 2007 | 2002 |  |  |  |  |
| Brazil | Costa | 2010 | 2004 |  |  |  |  |
| Brazil | Carnelosso | 2010 | 2004 |  |  |  |  |
| Brazil | Cabral | 2015 | 2013 | 182.43 | 110.76 | 51.31 | 101.62 |
| Brazil | Matos | 2003 |  | 205 | 116.7 | 70 | 128.6 |
| Chile | Jadue | 1999 | 1997 |  |  |  |  |
| Chile | Garcia Hermozo | 2017 | 2009 | 194.7 |  |  | 149.9 |
| Chile | Vinueza | 2010 | 2004 | 199.1 | 119.6 | 49.4 | 159.6 |
| Chile | Palomo | 2007 | 2005 |  |  |  |  |
| Chile | Tejos | 2013 | 2010 | 189.05 | 113.43 | 47.23 | 144.07 |
| Chile | Villanueva | 2018 | 2010 |  |  |  |  |
| Chile | Rubinstein | 2015 | 2011 | 202 | 125.6 | 45 | 169.1 |
| Chile | Lanas | 2016 | 2009 | 189.1 | 113.78 | 47.82 |  |
| Chile | Lara | 2012 |  | 178.3 | 114.6 | 41.4 | 112.5 |
| Chile | Mena | 2015 |  | 196.8 | 110.1 | 52.1 | 161.3 |
| Chile | Miquel | 1998 |  | 184.35 |  | 44.92 | 126.09 |
| Chile | Mujica | 2008 |  |  |  | 51.9 | 162.01 |
| Chile | Valenzuela | 2010 | 2003 |  |  |  |  |
| Chile | Group I multicentre collaborative | 1992 | 1989 | 193.35 |  | 38.67 |  |
| Chile | Cuevas | 2008 | 1997 |  |  |  |  |
| Chile | Pivatto | 2007 | 2001 |  |  |  |  |
| Chile | Amigo | 2010 | 2002 | 176.72 | 113.69 | 40.99 | 111.6 |
| Chile | LabraÃ±a | 2017 | 2009 |  |  |  |  |
| Chile | Acevedo | 2009 | 2007 | 201.3 | 120.6 | 49.5 | 156.4 |
| Chile | Acevedo | 2012 | 2005 | 202 | 121 | 50 | 157 |
| Chile | Berrios | 1997 | 1988 | 195.84 |  |  |  |
| Colombia | Roldan-Menco | 2017 |  | 202.66 | 115.87 | 41.04 | 194.45 |
| Colombia | Pirkle | 2018 | 2012 |  |  | 48 | 149 |
| Colombia | Arbey | 2011 |  | 202 | 119.5 | 42.8 | 210 |
| Colombia | Patino-Villada | 2011 | 2009 |  |  |  |  |
| Colombia | Camacho | 2018 | 2006 | 201.1 | 118.9 | 41.9 | 181.5 |
| Colombia | Vinueza | 2010 | 2004 | 193.7 | 120.4 | 42.2 | 164.7 |
| Colombia | Palmett-Rios | 2017 | 2011 |  |  | 39.11 |  |
| Colombia | Bautista | 2006 | 2001 | 200.31 | 134.96 | 39.83 |  |
| Colombia | Gallo | 2013 | 2007 | 222.18 | 149.4 | 39.78 | 163.3 |
| Colombia | AlayÃ³n | 2010 | 2008 |  |  |  |  |
| Costa Rica | Campos | 1991 | 1988 |  |  |  |  |
| Costa Rica | Wong-McClure | 2015 | 2005 |  |  |  |  |
| Costa Rica | Holst | 2006 | 2001 | 208.98 | 135.8 | 39.1 | 173.7 |
| Costa Rica | Chanti-Ketterl | 2017 | 2005 | 216.55 |  |  |  |
| Costa Rica | Campos | 1991 | 1988 | 180.7 | 110.72 | 42.83 | 134.8 |
| Costa Rica | Rehkopf | 2018 | 2005 |  |  | 46 | 170 |
| Costa Rica | Jimenez | 1987 | 1982 | 203.77 |  |  | 128.31 |
| Costa Rica | Goldman | 2011 | 2005 |  |  |  |  |
| Costa Rica | Williams | 2007 | 1996 | 201.31 |  | 41.95 | 209.44 |
| Cuba | Salas | 2016 | 2004 |  |  |  |  |
| Cuba | Nordet | 2013 | 2009 | 181.75 |  |  |  |
| Dominica | Robinson | 2004 |  | 182.4 | 105.9 | 49 | 133.4 |
| Dominican Republic | Aono | 1999 | 1993 | 177 |  | 40.7 |  |
| Dominican Republic | Salas | 2016 | 2005 |  |  |  |  |
| Dominican Republic | Dong | 2011 | 1997 | 183.5 | 109.5 | 50.3 | 119.2 |
| Ecuador | Encalada-Torres | 2017 | 2015 |  |  | 40.65 | 158.42 |
| Ecuador | Vinueza | 2010 | 2004 | 207.3 | 126.6 | 49 | 162.5 |
| Ecuador | Orces | 2017 | 2010 |  |  |  |  |
| Ecuador | Sisa | 2018 | 2010 | 200.2 | 117.33 | 48.3 | 141.67 |
| El Salvador | Orantes | 2011 | 2009 |  |  |  |  |
| Granada | Bansilal | 2012 | 2009 | 176.88 | 115.43 | 48.14 |  |
| Guatemala | Wong-McClure | 2015 | 2005 |  |  |  |  |
| Guatemala | Romero-Abal | 1994 |  | 220.8 |  |  | 195.2 |
| Guatemala | Cria O Gregory | 2009 | 1998 | 141.15 |  | 37.12 | 129.32 |
| Haiti | DeGennaro | 2018 | 2016 |  |  |  |  |
| Honduras | Wong-McClure | 2015 | 2005 |  |  |  |  |
| Honduras | Jaime Hall MartÃ­ne | 2005 | 2003 |  | 98.71 | 43.42 | 228.23 |
| Jamaica | Ferguson | 2017 | 2007 | 172.47 |  |  |  |
| Jamaica | Gupta | 2010 | 1996 | 184.46 | 121.42 | 50.27 | 71.74 |
| Jamaica | Florey | 1973 |  | 216.72 |  |  | 79.82 |
| Jamaica | Ferguson | 2008 | 2001 | 174.01 |  |  |  |
| Jamaica | Ferguson | 2010 | 2006 |  |  | 46.4 | 51.37 |
| Jamaica | Tulloch-Reid1 | 2013 | 2008 |  |  |  |  |
| Jamaica | Stringhini | 2016 | 2011 |  |  |  |  |
| Mexico | Vinueza | 2010 | 2004 | 202.9 | 118.7 | 49.2 | 183.9 |
| Mexico | Rosas-Saucedo | 2009 |  |  |  |  |  |
| Mexico | Sanchez-Corona | 2004 |  | 185.62 |  |  | 158.54 |
| Mexico | Aguilar-Salinas | 2002 | 1993 | 174.05 |  | 37.75 | 199.37 |
| Mexico | Aguilar-Salinas | 2011 | 2000 | 196.4 | 112.53 | 43.7 | 201.5 |
| Mexico | Escobedo de la PeÃ±a | 2014 | 2004 | 202.9 | 118.7 | 49.2 | 183.9 |
| Mexico | EchevarrÃ­a-Pinto | 2006 | 2005 |  |  |  |  |
| Mexico | Salas | 2016 | 2007 |  |  |  |  |
| Mexico | Rodriguez-Moran | 2001 |  | 209.98 | 135.34 | 41.38 | 404.76 |
| Mexico | Rodriguez-Ramirez | 2015 |  | 202.1 |  | 43.6 | 172.6 |
| Mexico | Posadas-Romero | 1995 | 1987 | 185.83 |  |  |  |
| Mexico | Valdez | 1995 | 1991 | 190.76 | 123.28 | 32.95 | 176.43 |
| Mexico | Kumar | 2016 | 2001 |  |  |  |  |
| Mexico | Hernandez | 2017 | 2012 | 193 |  |  | 181.5 |
| Mexico | Posadas-Romero | 1994 | 1991 | 206.88 | 138.44 | 43.31 | 157.65 |
| Mexico | Aguilar | 1999 |  | 165 |  | 42 | 126 |
| Mexico | Ferrannini | 2009 |  |  | 118.02 | 33.37 | 211.42 |
| Mexico | Aguilar-Salinas | 2010 | 2006 | 198.5 | 131.5 | 38.9 | 139.6 |
| Mexico | Gonzales-Villalpando | 1999 |  |  | 122.74 | 32.83 | 210.96 |
| Nicaragua | Wong-McClure | 2015 | 2005 |  |  |  |  |
| Peru | Salas | 2016 | 2006 |  |  |  |  |
| Peru | Seclen | 2006 | 2000 |  |  | 54.14 | 144.37 |
| Peru | Soto | 2005 | 2004 | 203.6 |  | 48.1 | 161 |
| Peru | Vinueza | 2010 | 2004 | 188.4 | 121.5 | 39.4 | 140.3 |
| Peru | Benzinger | 2010 | 2005 |  |  |  |  |
| Peru | Quispe | 2016 | 2011 |  |  |  |  |
| Peru | Baracco | 2007 | 2002 |  |  | 53.83 | 156.41 |
| Peru | Benziger | 2018 | 2010 |  |  |  |  |
| Peru | Benziger | 2015 | 2010 |  |  | 41.49 | 160.62 |
| Peru | Chirinos | 2014 |  |  | 115.18 | 46.18 | 152.35 |
| Peru | Seclen | 1999 |  |  |  |  |  |
| Peru | Goldstein | 2005 | 1999 |  |  |  |  |
| Peru | Medina-Lezama | 2007 | 2005 | 201.52 |  |  |  |
| Peru | Miranda | 2011 | 2007 | 184.49 | 110.53 | 44.1 | 129.34 |
| Peru | Malaga | 2010 | 2009 |  |  |  |  |
| Peru | Gaziano | 2016 | 2010 | 200.2 |  |  |  |
| Peru | Gonzales | 2013 | 2010 | 170.18 | 102.62 | 36.61 | 182.63 |
| Peru | Cardenas | 2009 | 2005 |  |  |  |  |
| Puerto Rico | Garcia Palmieri | 1972 |  | 202.63 |  |  | 149.9 |
| Puerto Rico | Perez | 2011 | 2006 | 191.3 | 117.5 | 49.4 | 141.7 |
| Puerto Rico | Salas | 2016 | 2008 |  |  |  |  |
| Puerto Rico | Cruz-Vidal | 1979 | 1964 | 201.41 |  |  | 146.06 |
| Puerto Rico | Castelli | 1977 | 1965 | 188.78 |  |  | 155.19 |
| Puerto Rico | Costas | 1978 | 1965 | 191 |  |  | 164 |
| Surinam | Krishnadath | 2016 | 2013 | 170.14 | 116.1 | 46.4 | 115.15 |
| Trinidad and Tobago | Miljkovic-Gacic | 2006 |  | 179.2 | 113.7 | 49.9 | 78.7 |
| Trinidad and Tobago | Miller | 1989 | 1979 | 228.93 | 162.41 | 42.54 |  |
| Uruguay | Rubinstein | 2015 | 2011 | 208.3 | 133 | 48.2 | 140.3 |
| US Virgin Islands | Tull | 2005 | 1998 |  |  |  |  |
| US Virgin Islands | Tull | 2013 | 1998 |  |  | 48.72 | 220.54 |
| Venezuela | Uzcategui | 2015 | 2006 |  |  |  |  |
| Venezuela | Salazar | 2018 | 2008 |  |  |  |  |
| Venezuela | Bermudez | 2017 | 2014 |  |  |  |  |
| Venezuela | Fernandez | 2006 |  | 178.29 |  | 44.4 | 157.17 |
| Venezuela | Florez | 2005 | 2000 | 170.74 |  | 43.42 | 135.7 |
| Venezuela | Gonzales-Rivas | 2016 | 2008 | 206.92 | 131.18 | 45.88 | 154.19 |
| Venezuela | Becerra | 2009 | 2007 | 185 |  | 41 | 184 |
| Venezuela | Vinueza | 2010 | 2004 | 174.2 | 104.6 | 40.1 | 150.7 |
| Venezuela | Salas | 2016 | 2006 |  |  |  |  |
| Venezuela | Bermudez | 2013 | 2013 | 189.27 | 119.86 | 44.35 | 127.53 |

# Supplementary Table 3: prevalence estimates of selected lipid biomarkers (197 studies)

| **Country** | **1st author** | **Publication year** | **Data collection year** | **Total cholesterol** | | **LDL-cholesterol** | | **HDL-cholesterol** | | **Triglycerides** | |
| --- | --- | --- | --- | --- | --- | --- | --- | --- | --- | --- | --- |
| Argentina | Vinueza | 2010 | 2004 | 18.7 | >=240 | 24.7 | mixed with Fr | 16.9 | <40 | 9.8 | >=200 |
| Argentina | Salazar | 2013 | 2003 |  |  |  |  |  |  |  |  |
| Argentina | Rubinstein | 2015 | 2011 | 21.75 | >=240 | 20.35 | >=160 | 35.49 | <40 | 21.25 | >=200 |
| Argentina | Salazar | 2009 | 2007 |  |  |  |  |  |  |  |  |
| Argentina | Carbajal | 2001 |  | 18.58 | >=5.18 |  |  |  |  |  |  |
| Argentina | Schneider | 2006 | 2001 |  |  |  |  |  |  |  |  |
| Argentina | Ferrante | 2007 | 2005 | 27.8 |  |  |  |  |  |  |  |
| Argentina | Redruello | 2008 | 2007 |  |  |  |  |  |  |  |  |
| Argentina | Ferrante | 2011 | 2009 | 29.1 |  |  |  |  |  |  |  |
| Barbados | Howitt | 2015 | 2012 | 21.2 | >=194 |  |  |  |  |  |  |
| Belize | Wong-McClure | 2015 | 2005 |  |  |  |  | 51 | <40(men) & <50 (women) | 38 | >=150 |
| Brazil | Velasquez-Melendez | 1999 | 1991 |  |  |  |  |  |  |  |  |
| Brazil | Pereira | 2006 | 2000 |  |  |  |  |  |  |  |  |
| Brazil | Poletto | 1992 | 1989 |  |  |  |  |  |  |  |  |
| Brazil | Rigo | 2008 | 2005 |  |  |  |  |  |  |  |  |
| Brazil | Sesso | 2008 | 1992 |  |  |  |  |  |  |  |  |
| Brazil | Pimenta | 2013 | 2005 |  |  |  |  | 44.1 | <40(men) & <50 (women) | 15.2 | >=150 |
| Brazil | Quintino-Santos | 2012 | 1996 |  |  |  |  |  |  |  |  |
| Brazil | Togeiro | 2013 | 2007 |  |  |  |  |  |  |  |  |
| Brazil | Ota | 2011 | 2001 |  |  |  |  |  |  |  |  |
| Brazil | Pimenta | 2007 | 2001 | 47.45 | >=200 | 43.14 | >=130 | 20.63 | <40 | 8.17 | >=200 |
| Brazil | Souza | 2003 | 2001 | 4.2 | >=240 | 13.1 | >=130 | 14.3 | <40 | 28.2 | >=150 |
| Brazil | Yokota | 2012 | 2010 | 49.2 | >200 | 48.4 | >200 | 36.9 | <40 | 10 | >200 |
| Brazil | Petris | 2016 | 2011 | 18.6 | >=240 | 17.31 | >=160 | 31.3 | <40 | 20.06 | >=200 |
| Brazil | Pirkle | 2018 | 2012 |  |  |  |  |  |  |  |  |
| Brazil | Werle | 2011 | 1994 |  |  |  |  |  |  |  |  |
| Brazil | Oliveira | 2012 | 2006 | 13 | >=200 |  |  |  |  | 39.7 | >=150 |
| Brazil | Venturini | 2013 | 2006 | 56.94 | >200 | 21.56 | >=160 | 59.4 | <40(men) & <50 (women) | 49.77 | >=150 |
| Brazil | Pavan | 1997 |  |  |  |  |  |  |  |  |  |
| Brazil | Roriz-Cruz | 2007 |  |  |  |  |  | 52.65 | <40(men) & <45 (women) | 67.52 | >=150 |
| Brazil | Neumann | 2007 | 2001 | 35.5 | >=200 | 34.5 | >=100 | 43.4 | <40 | 26.6 | >=150 |
| Brazil | Nunes Filho | 2007 | 2006 | 8.5 | >240 | 3.3 | >160 | 7.1 | <40 |  |  |
| Brazil | Gimeno | 2011 | 2006 | 22.8 | >200 |  |  |  |  |  |  |
| Brazil | Moraes | 2012 | 2007 | 43.7 | >=200 | 33.3 | >=130 | 20.4 | <40 | 25 | >=150 |
| Brazil | Garcez | 2014 | 2008 | 27.09 | >=200 |  |  | 44.15 | <=40(men) & <=50 (women) |  |  |
| Brazil | Garcez | 2014 | 2008 | 36.5 | >=200 |  |  | 30.04 | <=40(men) & <=50 (women) |  |  |
| Brazil | Igor Conterato Gomes | 2013 | 2010 | 51.3 | >200 | 31 | >150 |  |  |  |  |
| Brazil | Fortanelli | 2018 | 2015 | 17.15 | >=240 | 15.42 | >=160 |  |  |  |  |
| Brazil | Marquezine | 2008 |  | 59.5 | >=200 | 57.9 | >=130 | 54.3 | <40(men) & <50 (women) | 30.9 | >=150 |
| Brazil | Mendes-Lana | 2007 |  |  |  |  |  |  |  |  |  |
| Brazil | Pena | 2016 | 2009 |  |  |  |  |  |  |  |  |
| Brazil | Bieleman | 2014 | 1982 |  |  |  |  | 15.1 | <40 | 22.6 | >=150 |
| Brazil | Cardoso | 2002 | 1991 | 35.6 | >=200 | 34.1 | >=130 | 82 | <35 | 13 | >=200 |
| Brazil | Buffarini | 2018 | 1993 |  |  |  |  |  |  |  |  |
| Brazil | de Oliveira | 2010 | 1999 |  |  |  |  |  |  |  |  |
| Brazil | Martins | 1989 | 1987 | 19.71 | >200 |  |  | 37.63 | <35(men) & <45 (women) | 19 | >140 |
| Brazil | Fornes | 2000 | 1990 |  |  | 12 | >=160 | 22.1 | <=35 |  |  |
| Brazil | Martins | 1996 | 1991 |  |  |  |  |  |  |  |  |
| Brazil | Correa Leite | 2013 | 1997 |  |  |  |  |  |  |  |  |
| Brazil | Gus | 2002 | 2000 | 5.6 | >=240 |  |  |  |  |  |  |
| Brazil | Marcopito | 2005 | 2001 | 8.1 | >=240 |  |  | 27.1 | <40 | 14.4 | >=200 |
| Brazil | de Oliveira | 2008 | 2006 | 34.5 | >=200 | 25.7 | >=130 | 29.3 | <40(men) & <50 (women) | 31.1 | >=130 |
| Brazil | de Castro | 2015 | 2009 |  |  | 30.5 | >=130 | 38 | <40(men) & <50 (women) | 30.5 | >=150 |
| Brazil | de Souza | 2016 | 2009 |  |  |  |  |  |  |  |  |
| Brazil | Drumond | 2011 | 2009 | 51 | >=200 | 17.8 | >=160 | 34.6 | <40(men) & <50 (women) | 17.3 | >=150 |
| Brazil | Mendes | 2014 | 2009 | 19.41 | >=240 |  |  |  |  |  |  |
| Brazil | Castro | 2016 | 2010 |  |  | 38 |  | 37 |  | 32 |  |
| Brazil | Cocate | 2013 | 2011 |  |  |  |  |  |  |  |  |
| Brazil | Costa-Fagundes | 2018 | 2011 | 52.4 | >=200 |  |  |  |  |  |  |
| Brazil | Barbosa | 2016 | 2012 |  |  |  |  | 61.2 | <40(men) & <50 (women) | 17.3 | >150 |
| Brazil | Bernardi | 2015 | 2003 |  |  |  |  |  |  |  |  |
| Brazil | Candido | 2007 |  |  |  |  |  |  |  |  |  |
| Brazil | de Oliveira | 2012 |  |  |  |  |  |  |  |  |  |
| Brazil | de Oliveira | 2013 |  |  |  |  |  |  |  |  |  |
| Brazil | de Oliveira | 2017 |  |  |  |  |  |  |  |  |  |
| Brazil | Dressler | 2006 |  |  |  |  |  |  |  |  |  |
| Brazil | Beleigoli | 2017 | 2000 |  |  |  |  |  |  |  |  |
| Brazil | Bustos | 2007 | 2002 |  |  |  |  | 42.2 | <40(men) & <50 (women) | 12.9 | >=150 |
| Brazil | Costa | 2010 | 2004 | 23.6 |  |  |  |  |  |  |  |
| Brazil | Carnelosso | 2010 | 2004 | 44.4 | >200 |  |  |  |  | 13.3 | >=200 |
| Brazil | Cabral | 2015 | 2013 |  |  |  |  |  |  |  |  |
| Brazil | Matos | 2003 |  | 51.6 | >=200 | 14.3 | >=160 |  |  | 14.3 | >=200 |
| Chile | Jadue | 1999 | 1997 | 46.9 | >=200 |  |  |  |  |  |  |
| Chile | Garcia Hermozo | 2017 | 2009 | 43.3 | >=200 |  |  |  |  |  |  |
| Chile | Vinueza | 2010 | 2004 | 15.3 | >=240 | 19.9 | mixed with Fr | 21.2 | <40 | 22.7 | >=200 |
| Chile | Palomo | 2007 | 2005 | 44.5 | >200 | 65.3 | >100 | 39.2 | <40(men) & <50 (women) | 40.1 | >=150 |
| Chile | Tejos | 2013 | 2010 |  |  |  |  |  |  |  |  |
| Chile | Villanueva | 2018 | 2010 | 38.46 | >=200 | 30.23 | >=130 | 28.91 | <40(men) & <50 (women) | 30.75 | >=150 |
| Chile | Rubinstein | 2015 | 2011 | 24.8 | >=240 | 22.9 | >=160 | 36.9 | <40 | 25.7 | >=200 |
| Chile | Lanas | 2016 | 2009 | 38.5 | >200 | 21.9 | increased |  | <40 |  |  |
| Chile | Lara | 2012 |  | 25.9 | >=200 | 10.4 | >=160 | 49 | <=40 | 17.6 | >=150 |
| Chile | Mena | 2015 |  |  |  |  |  |  |  |  |  |
| Chile | Miquel | 1998 |  |  |  |  |  |  |  |  |  |
| Chile | Mujica | 2008 |  |  |  |  |  | 21.5 | <40(men) & <50 (women) | 40.1 | >=150 |
| Chile | Valenzuela | 2010 | 2003 |  |  |  |  | 52.6 | <40(men) & <50 (women) | 30 | >=150 |
| Chile | Group I multicentre collaborative | 1992 | 1989 |  |  |  |  |  |  |  |  |
| Chile | Cuevas | 2008 | 1997 | 37.3 | >=200 |  |  |  |  |  |  |
| Chile | Pivatto | 2007 | 2001 |  |  |  |  | 66.7 | <40(men) & <50 (women) | 17.9 | >=150 |
| Chile | Amigo | 2010 | 2002 |  |  |  |  |  |  |  |  |
| Chile | LabraÃ±a | 2017 | 2009 |  | >=200 |  |  | 47.3 | <40(men) & <50 (women) | 35.2 | >=150 |
| Chile | Acevedo | 2009 | 2007 |  |  |  |  |  |  |  |  |
| Chile | Acevedo | 2012 | 2005 |  |  |  |  |  |  |  |  |
| Chile | Berrios | 1997 | 1988 | 7.23 | >=6.15 |  |  |  |  |  |  |
| Colombia | Roldan-Menco | 2017 |  |  |  |  |  | 79.6 | <40(men) & <50 (women) | 75.9 | >150 |
| Colombia | Pirkle | 2018 | 2012 |  |  |  |  |  |  |  |  |
| Colombia | Arbey | 2011 |  |  |  |  |  |  |  |  |  |
| Colombia | Patino-Villada | 2011 | 2009 | 9 | >=240 | 5.9 | >=160 | 14 | <=40 | 19.3 | >=200 |
| Colombia | Camacho | 2018 | 2006 | 48.7 | >=200 | 75.3 | >=130 | 57.1 | <=40(men) & <=50 (women) | 49.7 | >=150 |
| Colombia | Vinueza | 2010 | 2004 | 11.7 | >=240 | 19.1 | mixed with Fr | 45.6 | <40 | 23.2 | >=200 |
| Colombia | Palmett-Rios | 2017 | 2011 |  |  |  |  | 80.5 | <40(men) & <50 (women) |  |  |
| Colombia | Bautista | 2006 | 2001 | 18.3 | >=6.15 | 22.3 | >=4.1 | 27.7 | <0.9 |  |  |
| Colombia | Gallo | 2013 | 2007 |  |  |  |  |  |  |  |  |
| Colombia | AlayÃ³n | 2010 | 2008 | 39 | >200 | 34 | >130 | 42 | <40 | 39 |  |
| Costa Rica | Campos | 1991 | 1988 | 8.21 | >=240 | 8.32 | >=160 | 21.34 | <35 |  |  |
| Costa Rica | Wong-McClure | 2015 | 2005 |  |  |  |  | 62 | <40(men) & <50 (women) | 58 | >=150 |
| Costa Rica | Holst | 2006 | 2001 |  |  |  |  |  |  |  |  |
| Costa Rica | Chanti-Ketterl | 2017 | 2005 |  |  |  |  |  |  |  |  |
| Costa Rica | Campos | 1991 | 1988 |  |  |  |  |  |  |  |  |
| Costa Rica | Rehkopf | 2018 | 2005 |  |  |  |  |  |  |  |  |
| Costa Rica | Jimenez | 1987 | 1982 |  |  |  |  |  |  |  |  |
| Costa Rica | Goldman | 2011 | 2005 | 29.81 | >=240 |  |  |  |  | 23.61 | >=200 |
| Costa Rica | Williams | 2007 | 1996 |  |  |  |  | 60.36 | <40(men) & <50 (women) | 67.18 | >=150 |
| Cuba | Salas | 2016 | 2004 |  |  |  |  | 55.1 | <40(men) & <45 (women) | 32.7 | >=150 |
| Cuba | Nordet | 2013 | 2009 | 5.9 | >=6 |  |  |  |  |  |  |
| Dominica | Robinson | 2004 |  |  |  |  |  |  |  |  |  |
| Dominican Republic | Aono | 1999 | 1993 |  |  |  |  |  | Rep. Dom |  |  |
| Dominican Republic | Salas | 2016 | 2005 | 41.1 | >=5.2 |  |  |  |  | 19.4 | >=150 |
| Dominican Republic | Dong | 2011 | 1997 |  |  |  |  |  |  |  |  |
| Ecuador | Encalada-Torres | 2017 | 2015 |  |  |  |  |  |  |  |  |
| Ecuador | Vinueza | 2010 | 2004 | 20.2 | >=240 | 23.9 | mixed with Fr | 21.6 | <40 | 23.8 | >=200 |
| Ecuador | Orces | 2017 | 2010 | 16.54 | >240 | 12.24 | >=160 | 28.61 | <40 | 23.28 | >=200 |
| Ecuador | Sisa | 2018 | 2010 |  |  |  |  |  |  |  |  |
| El Salvador | Orantes | 2011 | 2009 | 23.2 | >240 | 15 | >160 | 19.6 | <35(men) & <39 (women) | 49.7 | >150 |
| Granada | Bansilal | 2012 | 2009 | 8.6 | >=240 |  |  |  |  |  |  |
| Guatemala | Wong-McClure | 2015 | 2005 |  |  |  |  | 38 | <40(men) & <50 (women) | 62 | >=150 |
| Guatemala | Romero-Abal | 1994 |  | 26 | >=240 |  |  |  |  | 36 | >170 |
| Guatemala | Cria O Gregory | 2009 | 1998 | 3.53 | >200 |  |  | 81.03 | <40(men) & <50 (women) | 28.73 | >=150 |
| Haiti | DeGennaro | 2018 | 2016 | 2.3 | >=240 | 2.7 | >=160 | 34.7 | <40(men) & <50 (women) | 7.4 | >=200 |
| Honduras | Wong-McClure | 2015 | 2005 |  |  |  |  | 21 | <40(men) & <50 (women) | 56 | >=150 |
| Honduras | Jaime Hall MartÃ­ne | 2005 | 2003 |  |  |  |  |  |  |  |  |
| Jamaica | Ferguson | 2017 | 2007 | 18.1 | >=5.2 |  |  |  |  |  |  |
| Jamaica | Gupta | 2010 | 1996 |  |  |  |  |  |  |  |  |
| Jamaica | Florey | 1973 |  |  |  |  |  |  |  |  |  |
| Jamaica | Ferguson | 2008 | 2001 |  |  |  |  |  |  |  |  |
| Jamaica | Ferguson | 2010 | 2006 |  |  |  |  |  |  |  |  |
| Jamaica | Tulloch-Reid1 | 2013 | 2008 | 18.4 | >=5.2 |  |  |  |  |  |  |
| Jamaica | Stringhini | 2016 | 2011 | 12.5 | >=5.2 |  |  |  |  |  |  |
| Mexico | Vinueza | 2010 | 2004 | 16.4 | >=240 | 25.6 | mixed with Fr | 22.6 | <40 | 32.5 | >=200 |
| Mexico | Rosas-Saucedo | 2009 |  | 34.3 |  |  |  |  |  | 45 |  |
| Mexico | Sanchez-Corona | 2004 |  |  |  |  |  |  |  |  |  |
| Mexico | Aguilar-Salinas | 2002 | 1993 |  |  |  |  |  |  |  |  |
| Mexico | Aguilar-Salinas | 2011 | 2000 |  |  |  |  |  |  |  |  |
| Mexico | Escobedo de la PeÃ±a | 2014 | 2004 | 16.4 | >=240 | 12.3 | >=160 | 22.6 | <40 | 32.5 | >=200 |
| Mexico | EchevarrÃ­a-Pinto | 2006 | 2005 |  |  |  |  |  |  | 61.6 | >=150 |
| Mexico | Salas | 2016 | 2007 |  |  |  |  | 44.39 | <40(men) & <45 (women) | 48.3 | >=150 |
| Mexico | Rodriguez-Moran | 2001 |  |  |  |  |  |  |  |  |  |
| Mexico | Rodriguez-Ramirez | 2015 |  |  |  |  |  |  |  |  |  |
| Mexico | Posadas-Romero | 1995 | 1987 | 10.6 | >=6.2 |  |  |  |  |  |  |
| Mexico | Valdez | 1995 | 1991 |  |  |  |  |  |  |  |  |
| Mexico | Kumar | 2016 | 2001 | 40.95 | >=200 |  |  | 69.25 | <40(men) & <50 (women) |  |  |
| Mexico | Hernandez | 2017 | 2012 | 37.3 | >=200 |  |  |  |  | 64.9 | >=150 |
| Mexico | Posadas-Romero | 1994 | 1991 |  |  |  |  |  |  |  |  |
| Mexico | Aguilar | 1999 |  |  |  |  |  |  |  |  |  |
| Mexico | Ferrannini | 2009 |  |  |  |  |  |  |  |  |  |
| Mexico | Aguilar-Salinas | 2010 | 2006 | 43.6 | >=200 | 46 | >=130 | 60.5 | <40 | 31.5 | >=150 |
| Mexico | Gonzales-Villalpando | 1999 |  |  |  |  |  |  |  |  |  |
| Nicaragua | Wong-McClure | 2015 | 2005 |  |  |  |  | 57 | <40(men) & <50 (women) | 42 | >=150 |
| Peru | Salas | 2016 | 2006 | 47.8 | >=5.2 |  |  |  |  |  |  |
| Peru | Seclen | 2006 | 2000 |  |  |  |  | 65.2 | <40(men) & <45 (women) | 83.1 | >=150 |
| Peru | Soto | 2005 | 2004 | 47.3 | >=200 |  |  | 56.3 | <40(men) & <50 (women) | 43.4 | >=150 |
| Peru | Vinueza | 2010 | 2004 | 11.6 | >=240 | 17.7 | mixed with Fr | 56.9 | <40 | 19.5 | >=200 |
| Peru | Benzinger | 2010 | 2005 |  |  |  |  | 43.31 | <40(men) & <50 (women) |  |  |
| Peru | Quispe | 2016 | 2011 |  |  |  |  | 65.85 | <40(men) & <50 (women) | 43.1 | >=150 |
| Peru | Baracco | 2007 | 2002 |  |  |  |  |  |  |  |  |
| Peru | Benziger | 2018 | 2010 | 48.08 | >=200 |  |  |  |  |  |  |
| Peru | Benziger | 2015 | 2010 |  |  |  |  | 66.4 | <40(men) & <50 (women) | 43.1 | >=150 |
| Peru | Chirinos | 2014 |  |  |  |  |  |  |  |  |  |
| Peru | Seclen | 1999 |  | 26.66 | >=240 |  |  |  |  |  |  |
| Peru | Goldstein | 2005 | 1999 | 23.91 | >=240 |  |  | 39.02 | <40 |  |  |
| Peru | Medina-Lezama | 2007 | 2005 |  |  |  |  |  |  |  |  |
| Peru | Miranda | 2011 | 2007 |  |  |  |  |  |  |  |  |
| Peru | Malaga | 2010 | 2009 | 40.6 | >=200 | 71.6 | >=100 | 48.6 | <40 | 48.6 | >150 |
| Peru | Gaziano | 2016 | 2010 |  |  |  |  |  |  |  |  |
| Peru | Gonzales | 2013 | 2010 | 26.58 | >=200 | 11.23 | >=160 | 43.85 | <40 | 64.5 | >=150 |
| Peru | Cardenas | 2009 | 2005 |  | < 40 en Hombres y < 50 en mujeres |  |  | 54.2 | <40 | 30 | >=150 |
| Puerto Rico | Garcia Palmieri | 1972 |  |  |  |  |  |  |  |  |  |
| Puerto Rico | Perez | 2011 | 2006 |  |  |  |  | 45.8 | <40(men) & <50 (women) | 31.2 | >=150 |
| Puerto Rico | Salas | 2016 | 2008 |  |  |  |  | 47.6 | <40(men) & <45 (women) | 32.9 | >=150 |
| Puerto Rico | Cruz-Vidal | 1979 | 1964 |  |  |  |  |  |  |  |  |
| Puerto Rico | Castelli | 1977 | 1965 |  |  |  |  |  |  |  |  |
| Puerto Rico | Costas | 1978 | 1965 |  |  |  |  |  |  |  |  |
| Surinam | Krishnadath | 2016 | 2013 |  |  |  |  |  |  |  |  |
| Trinidad and Tobago | Miljkovic-Gacic | 2006 |  |  |  |  |  |  |  |  |  |
| Trinidad and Tobago | Miller | 1989 | 1979 |  |  |  |  |  |  |  |  |
| Uruguay | Rubinstein | 2015 | 2011 | 31 | >=240 | 31.6 | >=160 | 25.4 | <40 | 15.7 | >=200 |
| US Virgin Islands | Tull | 2005 | 1998 |  |  |  |  | 43.31 | <40(men) & <50 (women) | 14.66 | >=150 |
| US Virgin Islands | Tull | 2013 | 1998 |  |  |  |  |  |  |  |  |
| Venezuela | Uzcategui | 2015 | 2006 |  |  |  |  | 84.2 | <40(men) & <50 (women) | 35.7 | >=150 |
| Venezuela | Salazar | 2018 | 2008 |  |  |  |  |  |  | 27.2 | >=150 |
| Venezuela | Bermudez | 2017 | 2014 |  |  |  |  |  |  | 35 | >=150 |
| Venezuela | Fernandez | 2006 |  |  |  |  |  |  |  |  |  |
| Venezuela | Florez | 2005 | 2000 |  |  |  |  | 65.3 | <40(men) & <50 (women) | 32.3 | >=150 |
| Venezuela | Gonzales-Rivas | 2016 | 2008 |  |  |  |  |  |  |  |  |
| Venezuela | Becerra | 2009 | 2007 | 33 | >=200 |  |  | 76 | <50 | 56 | >=150 |
| Venezuela | Vinueza | 2010 | 2004 | 5.7 | >=240 | 9.8 | mixed with Fr | 52.2 | <40 | 20.2 | >=200 |
| Venezuela | Salas | 2016 | 2006 | 60.8 | >=5.2 |  |  |  |  | 43.5 | >=150 |
| Venezuela | Bermudez | 2013 | 2013 |  |  |  |  | 58 | <40(men) & <50 (women) | 26.6 | >=150 |

Definitions for prevalence estimates are in mg/dl or mmol/l, and as originally reported. For the random-effects meta-analysis, consistent and relevant metrics were pooled (i.e., other were not pooled but presented in the time trends results).

# Supplementary Table 4: Selected lipid clinical guidelines from Latin America and the Caribbean

Statin Eligibility Criteria According to 7 Major Latin American Primary Prevention Guidelines

|  | **Lipid-based (Cholesterol level)** | **Risk-based** | |
| --- | --- | --- | --- |
|  |  | **Age range, y** | **Eligibility** |
| **SUDEAT, Uruguay^1^** | LDL-C ≥190 mg/dL | NA | DM, LDL-C ≥160 mg/dL with 2 cardiovascular risk factors. |
| **CCSS, Costa Rica 2004^2^** | LDL-C ≥190 mg/dL, HDL-C < 40 mg/dL | Male ≥ 45, Female ≥ 55 | Hypertension (BP ≥ 140/90 mmHg or with antihypertensive treatment), history of early-onset coronary heart disease, first-degree male relative with history of coronary heart disease at age 55 or earlier, first-degree female relative with history of coronary heart disease at age 65 or earlier, smoking history.^a^ |
| **SBC, Brazil 2013^3^** | NA | NA | Familial hypercholesterolemia, CKD or microalbuminuria, metabolic syndrome according to IDF criteria, DM type 1 and type 2, family history of early-onset coronary heart disease,^g^ left ventricular hypertrophy, high sensitivity CRP > 2 mg/L, subclinical atherosclerosis based on diagnostic studies.^b^ |
| **CINETS, Colombia 2014^4^** | LDL-C >190 mg/dL | NA | FRS > 10% predicted 10-y risk of any ASCVD, diabetes mellitus, history of atherosclerotic cardiovascular disease.^c^ |
| **IMSS, Mexico 2016^5^** | LDL-C ≥190 mg/dL | 40-75 | Familial hypercholesterolemia, DM, LDL-C of 100-189 mg/dL with very high cardiovascular risk^d^, LDL-C of 155-189mg/dL with high cardiovascular risk,^e^ LDL-C of 155-189 mg/dL with moderate cardiovascular risk.^f^ |
| **SAC, Argentina 2018^6^** | LDL-C >190 mg/dL | NA | Familial hypercholesterolemia, moderate CKD (GFR 30-59 mL/min/1,73m^2^) or severe CKD (GFR less than 30 mL/min/1,73m^2^) without haemodialysis, DM, cardiovascular ≥20% predicted 10-y risk of any ASCVD, cardiovascular 10-19% predicted 10-y risk of any ASCVD with 1 risk factor or CRP > 2 mg/L, subclinical atheromatosis.^g^ |
| **MINSAL, Chile 2018^7^** | LDL-C >190 mg/dL | NA | DM, CKD stages 3b – 5 or albuminuria, resistant hypertension. |

Abbreviations: SUDEAT, Sociedad Uruguaya de Ateroesclerosis; CCSS, Caja Costarricense del Seguro social; SBC, Sociedade Brasileira de Cardiologia; CINETS, Centro Nacional de Investigación en Evidencia y Tecnologías en Salud; IMSS, Instituto Mexicano del Seguro social; SAC, Sociedad Argentina de Cardiología; MINSAL, Ministerio de Salud de Chile; FRS, Framingham Risk Score; IDF, International Diabetes Federation; ASCVD, atherosclerotic cardiovascular disease; CKD, chronic kidney disease; DM, diabetes mellitus; LDL-C, low-density lipoprotein colesterol; GFR, glomerular filtration rate; CRP, C-reactive protein.

^a^ For primary prevention: 0-1 risk factors.

^b^ Carotid intima-media thickness >1, coronary calcium score >100 or > 75^th^ percentile for sex and age, ankle-brachial index <0.9.

^c^ Acute coronary events such as acute myocardial infarction (AMI) and stable or unstable angina, cerebrovascular accident (CVA), transitory ischemic attack (TIA), history of previous revascularization (coronary or other site), or lower extremity atherosclerotic vascular disease.

^d^ Established cardiovascular disease by imaging, history of AMI, coronary revascularization or any other revascularization method, ischemic-type cerebrovascular event, DM type 2 or 1 with target organ damage, moderate to severe GFR decrease (< 60 ml/min/1.73 m^2^), Globorisk > 10% predicted 10-y risk of any ASCVD.

^e^ Globorisk 5-10% predicted 10-y risk of any ASCVD; markedly elevated risk factors such as uncontrolled blood pressure (blood pressure ≥180/10mmHg), uncontrolled dyslipidaemia (total cholesterol > 310mg/dL) or with primary familial hypercholesterolemia.

^f^ Globorisk 1-5% predicted 10-y risk of any ASCVD.

^g^ Significant peripheral arterial plaque (carotid, femoral or aortic), coronary calcium score > 75^th^ percentile or > 300 units Agatston, coronary plaque on angiotomography.

1. Sociedad Uruguaya de Aterosclerosis. Segundo consenso uruguayo sobre dislipidemias.
2. Caja Costarricense de Seguro social. Guías para la detección, el diagnóstico y el tratamiento de las dislipidemias para el primer nivel de atención; 2004
3. Xavier HT, Izar MC, Faria JR, Assad MH, Rocha VZ, Sposito AC, et al. V Diretriz Brasileira de Dislipidemias e Prevenção da Aterosclerose. Sociedade Brasileira de Cardiologia. 2013; 101(4)
4. Centro Nacional de Investigación en Evidencia y Tecnologías en Salud CINETS. Guía de práctica clínica para la prevención, detección temprana, diagnóstico, tratamiento y seguimiento de las dislipidemias en la población mayor de 18 años; 2014
5. Instituto Mexicano del Seguro Social. Diagnóstico y tratamiento de DISLIPIDEMIAS (HIPERCOLESTEROLEMIA) en el adulto; 2016
6. Sociedad Argentina de Cardiología. Uso apropiado de las estatinas en la Argentina, Documento de posición. Revista Argentina de Cardiología. 2018; 86(1)
7. Ministerio de Salud de Chile. Orientación técnica Dislipidemias; 2018

# Supplementary Table 5: Country classification into sub-regions

| **ISO3 Code** | **Country** | **Sub-Region** |
| --- | --- | --- |
| ARG | Argentina | Southern and Tropical Latin America |
| ATG | Antigua and Barbuda | Caribbean |
| BHS | Bahamas | Caribbean |
| BLZ | Belize | Caribbean |
| BMU | Bermuda | Caribbean |
| BOL | Bolivia | Andean Latin America |
| BRA | Brazil | Southern and Tropical Latin America |
| BRB | Barbados | Caribbean |
| CHL | Chile | Southern and Tropical Latin America |
| COL | Colombia | Central Latin America |
| CRI | Costa Rica | Central Latin America |
| CUB | Cuba | Caribbean |
| DMA | Dominica | Caribbean |
| DOM | Dominican Republic | Caribbean |
| ECU | Ecuador | Andean Latin America |
| GRD | Grenada | Caribbean |
| GTM | Guatemala | Central Latin America |
| GUY | Guyana | Caribbean |
| HND | Honduras | Central Latin America |
| HTI | Haiti | Caribbean |
| JAM | Jamaica | Caribbean |
| KNA | Saint Kitts and Nevis | Caribbean |
| LCA | Saint Lucia | Caribbean |
| MEX | Mexico | Central Latin America |
| NIC | Nicaragua | Central Latin America |
| PAN | Panama | Central Latin America |
| PER | Peru | Andean Latin America |
| PRI | Puerto Rico | Caribbean |
| PRY | Paraguay | Southern and Tropical Latin America |
| SLV | El Salvador | Central Latin America |
| SUR | Suriname | Caribbean |
| TTO | Trinidad and Tobago | Caribbean |
| URY | Uruguay | Southern and Tropical Latin America |
| VCT | Saint Vincent and the Grenadines | Caribbean |
| VEN | Venezuela | Central Latin America |

Classification based on NCD-RisC sub-regions: <http://ncdrisc.org/>

1. Hoy D, Brooks P, Woolf A, et al. Assessing risk of bias in prevalence studies: modification of an existing tool and evidence of interrater agreement. Journal of clinical epidemiology 2012; 65(9): 934-9. [↑](#footnote-ref-1)
